# An Integrative Approach Toward Bio‐Inspired Sequestration of Rare Earth Elements

**DOI:** 10.1002/cssc.70900

**Published:** 2026-08-12

**Authors:** Yulia Perevedentseva, Lukas Waurick, Dini Marlina, Gerda Techert, Yannic Müllers, Franziska Lederer, Heiko M. Möller, Michael U. Kumke, Ulrich Glebe, Björn Drobot

**Affiliations:** ^1^ Institute of Chemistry University of Potsdam Potsdam‐Golm Germany; ^2^ Institute of Resource Ecology Helmholtz‐Zentrum Dresden‐Rossendorf Dresden Germany; ^3^ Helmholtz Institute Freiberg for Resource Technology Helmholtz‐Zentrum Dresden‐Rossendorf Dresden Germany; ^4^ Fraunhofer Institute for Applied Polymer Research IAP Potsdam‐Golm Germany

**Keywords:** calmodulin, EF‐hand loop, lanthanide‐binding peptides, metal‐binding peptides, peptide dynamics

## Abstract

Lanthanides (Ln) are essential for renewable energy technologies, yet their extraction and recycling often cause severe environmental impacts. Bio‐inspired ligands based on metal‐binding protein motifs offer a promising alternative for selective and sustainable Ln recovery. This study investigates the lanthanide‐binding properties of the EF‐hand 4 (EF4) motif of calmodulin. The binding of various Ln^3+^ ions to EF4 was characterized using isothermal titration calorimetry, time‐resolved laser fluorescence spectroscopy, nuclear magnetic resonance spectroscopy, and molecular dynamics simulations. EF4 exhibits generally high affinity toward Ln, with slightly varying binding constants across the Ln series. Thermodynamic, structural, and coordination effects associated with Ln binding were analyzed in detail. Compared to the EF‐hand loop within the native protein, the isolated EF4 peptide shows significantly increased flexibility. Depending on experimental conditions, both 1:1 and 2:1 (Ln^3+^:peptide) complex stoichiometries were observed. Furthermore, immobilized EF4 was successfully applied for the extraction and recovery of Sm^3+^ from artificial aqueous solutions under laboratory conditions. Overall, the EF4 loop motif of calmodulin represents a promising biological ligand for the selective binding and recovery of Ln from aqueous systems.

## Graphical abstract

The binding behavior of EF‐hand 4 of calmodulin toward different lanthanide ions was studied using a combination of spectroscopic and computational methods. A resin‐immobilized peptide demonstrated its potential as a selective and sustainable filter material for lanthanide separation.

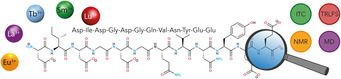

## 1 Introduction

Due to their special properties, lanthanides (Ln) have become indispensable in modern technology and are required for solar cells, light‐emitting diodes, magnets, and electronic devices, among others [1, 2]. The process of obtaining Ln through conventional mining is environmentally problematic. Ln have to be extracted from minerals such as monazite and bastnesite, which is accompanied by high energy consumption and emissions, thus being unsustainable [3]. Moreover, only a few countries hold the resources and infrastructure for mineral extraction [4]. Therefore, the development of sustainable processes for Ln recovery and recycling will be inevitable. Current recovery methods include pyrometallurgical [5], hydrometallurgical [6], biometallurgical, and electrochemical processes [7]. Pyrometallurgical and hydrometallurgical methods are the most widespread, although they are harmful to the environment. The processes typically involve the use of harsh chemicals or consume large amounts of energy, further increasing their negative environmental impact [8]. Biometallurgy represents an alternative to these processes, but it is difficult to control on an industrial scale as the used microorganisms are sensitive to operational conditions [9]. As a consequence of these limitations, recycling rates for Ln are still very low [10]. Developing and implementing more sustainable and eco‐friendly techniques for the extraction and recovery of Ln is crucial. New methods for extracting Ln are currently being actively researched. These include using membrane‐assisted solvent extraction (MSX) [11], carboxylic acid functionalized porous aromatic frameworks (PAFs) [12] and metal–organic frameworks (MOFs) [13, 14]. Furthermore, developing new separation processes involving biomolecules constitutes a promising approach. The mineralization concept for the direct extraction of Ln ions using Ln ion mineralization peptide has been shown [15, 16]. It was discovered that glutaraldehyde cross‐linking of Ln‐binding peptides enhances the adsorption of Ln to air–water interfaces and can be used for optimizing Ln recovery in foam‐based processes [17]. Furthermore, it was demonstrated that phage display is an effective tool for screening peptide libraries to identify high‐affinity binders for lanthanide compounds which are the components of electronic waste [18, 19, 20]. Ultimately, these results highlight the versatility of biomolecules in the development of next‐generation materials for Ln enrichment and separation. Beyond sequence optimization, rigidification through cyclization represents a further promising strategy to improve lanthanide‐binding peptides. The affinity can be tuned by imposing stoichiometric restrictions and pre‐structuring the binding site, as recently demonstrated for LanM‐derived peptides [21].

Many proteins contain metal‐binding sites with very high affinity and specificity for certain metal ions. For example, calmodulin (CaM) is a Ca^2+^ binding protein widely spread in all eukaryotic cells [22, 23, 24], and its homologs were also found in prokaryotic cells [25]. In addition, lanmodulin (LanM) was recently discovered in *Methylorubrum extorquens* AM1 as a “native” Ln binding protein [26, 27]. Both CaM and LanM possess four binding sites each with a helix‐loop‐helix motif, known as EF‐hand (EF1 to EF4) [28]. All EF‐hand loops are twelve amino acids long, and their sequences differ only slightly and contain negatively charged residues at conserved positions that are substantially involved in metal coordination (DxDGDGxxxxxE).

Remarkably, Ln can substitute ‐ at least to some extent ‐ for all Ca^2+^ in the four calcium‐binding sites [29, 30, 31, 32]. This property has been actively used in the study of calcium‐binding proteins since some Ln have additional spectroscopic properties that facilitate the analysis via luminescence spectroscopy [33]. Moreover, paramagnetic properties of some Ln have been exploited to provide deeper insights into the structure, dynamics, and interactions of proteins via nuclear magnetic resonance (NMR) spectroscopy [34, 35, 36]. The calmodulin EF‐hands have been engineered for improved affinity toward Ln and developed as a tool in chemical and structural biology research. This led to the well‐known lanthanide binding tag (LBT) (YIDTNNDGWYEGDELLA) that is used as a genetically encodable tag in protein NMR to provide a well‐defined Ln‐binding site for evoking paramagnetic relaxations enhancements (PRE), paramagnetism‐induced residual dipolar couplings (RDC) and pseudo contact shifts (PCS) [37, 38, 39, 40, 41, 42].

Using peptides instead of proteins as ligands for Ln binding would have several advantages such as circumventing the risk of denaturation, easier and more cost‐efficient production, and facilitated tailoring, functionalization, and immobilization to applicable devices. In this study, we chose the CaM‐EF‐hand 4 (EF4) as an exemplary peptide sequence to study its interaction with Ln ions. Among the four CaM EF‐hands, EF4 was selected because it contains five oxygen‐donor residues at relevant coordination positions (D1, D3, D5, E11, E12), matching the number found in the EF‐hand loops of lanmodulin, and thus representing the CaM EF‐hand with the highest potential for lanthanide coordination. Isothermal titration calorimetry (ITC), time‐resolved laser fluorescence spectroscopy (TRLFS), NMR spectroscopy, and molecular dynamics (MD) simulations provide detailed insight into the thermodynamics as well as structural and dynamic characteristics of the Ln^3+^‐EF4 complexes. To demonstrate its applicability for Ln recovery from aqueous solution, EF4 was immobilized on polymer beads and used for the extraction of Sm^3+^ ions. Overall, our study contributes to understanding how distinct, short peptides interact with Ln ions. The demonstrated extraction of samarium exemplifies the potential to develop sophisticated peptide‐based separation techniques in the future.

## 2 Experimental Section

Detailed information on the materials and methods used in this study is provided in the Supporting Information.

Five Ln were selected to cover complementary analytical requirements: Eu^3+^, Tb^3+^, and Sm^3+^ were chosen for their luminescence properties enabling TRLFS characterization, while La^3+^ and Lu^3+^ were selected as diamagnetic representatives at opposite ends of the lanthanide series, well‐suited for NMR studies without paramagnetic line broadening.

## 3 Results and Discussion

### 3.1 Thermodynamic Characterization: Affinity Determination for Eu^3+^, Sm^3+^, and Tb^3+^

**FIGURE 1:**
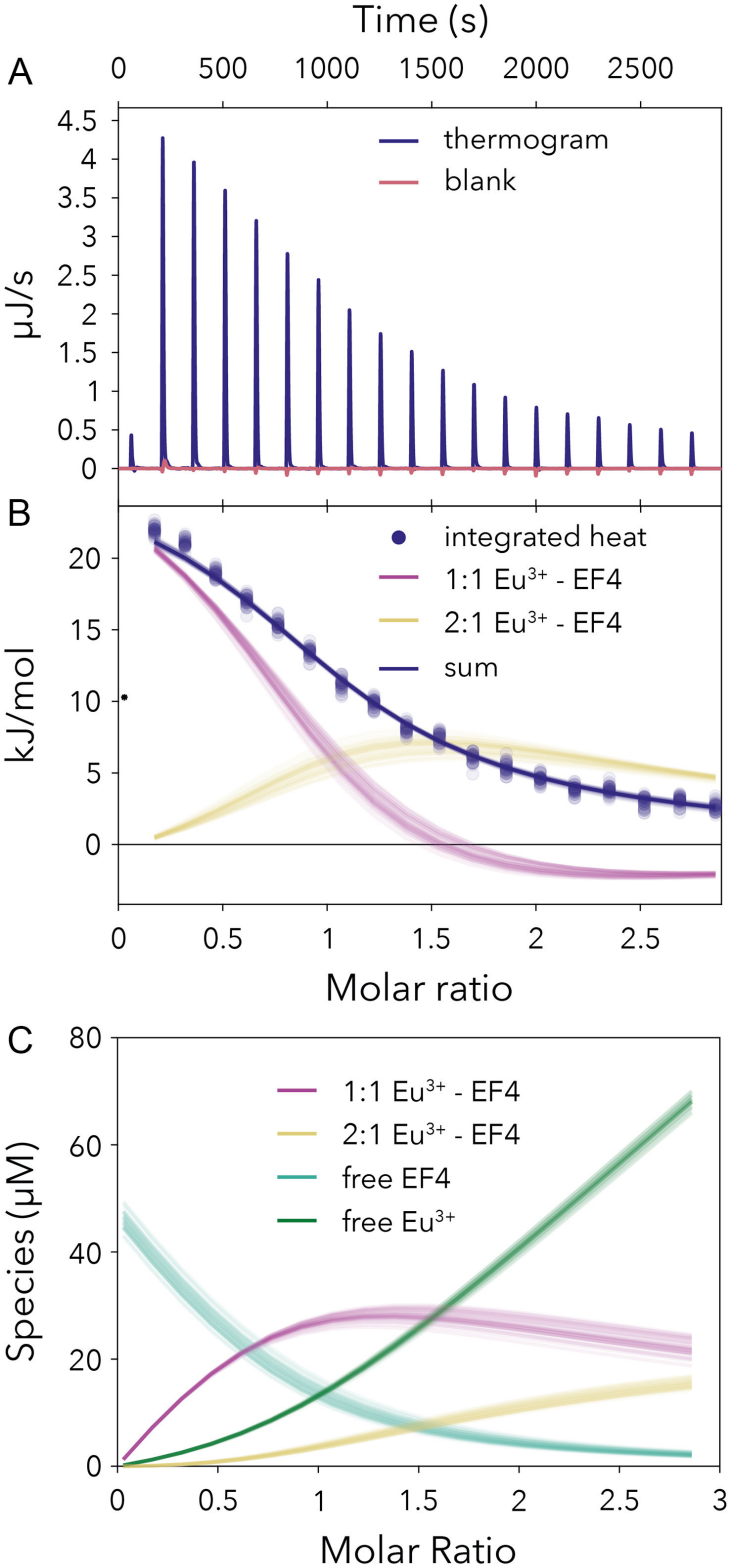
ITC of Eu^3+^ binding to EF4  (A). Baseline‐corrected thermograms of the titration of 750 µM Eu^3+^ to 50 µM EF4 (blue line) at pH 6.0 and the corresponding background measurement ‐ titration of Eu^3+^ into background electrolyte (100 mM KCl, pH 6.0; rose line) (B). Integrated heat of the thermogram (blue symbols). Transparent symbols are for the Monte Carlo approach for the estimation of the robustness of the fit and the estimation of the parameter standard deviation. Magenta and yellow curves are individual contributions of the 1:1 and 2:1 Eu^3+^‐EF4 complexes, and their sum is shown as blue lines. Underlying speciation (C). Experimental conditions and details on data acquisition and analysis are provided in the Supporting Information.

The binding studies were performed by ITC and TRLFS to compare the affinity of the three different Ln ions, Eu^3+^, Sm^3+^, and Tb^3+^, to the peptide in solution with the known affinity to CaM. Furthermore, ITC provides a thermodynamic analysis of the complex formation process. For that, we titrated 750 µM EuCl_3_ in three series and with 19 single injections each to different peptide concentrations, which were 100, 50 and 33 µM EF4, enabling us to identify differences in complex formation. The thermogram and the integrated heat plot are shown exemplarily for the Eu^3+^‐EF4 experiment in Figure 1. The binding reaction was found to be overall endothermic, which is consistent with the binding of Eu^3+^ to the protein CaM [43]. The stripping of the first and second hydration shells of the Ln^3+^ aquo ion constitutes the largest contribution to the measured energies, while the dehydration of the peptide might also play a role in the entropy increase (Table S1). Based on the baseline‐corrected thermogram, the most robust fit specified two complexes (Figure 1), where the 1:1 complex is the first dominant species, which gradually turns into a 2:1 Eu^3+^‐EF4 complex as secondary species with increasing Eu^3+^ concentration.

**FIGURE 2:**
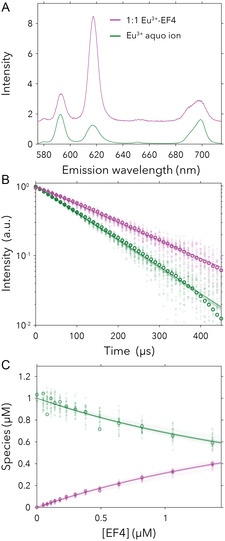
PARAFAC deconvolution of TRLFS data where 1 µM Eu^3+^ was titrated with 0–1.4 µM EF4. Luminescence spectra of each Eu^3+^ species (color code: green = free Eu^3+^ aquo ion, magenta = 1:1 Eu^3+^‐EF4) (A), luminescence decay kinetics (B), and corresponding species distribution (C). Experimental conditions and details on data acquisition and analysis are provided in the Supporting Information.

Complementary binding studies were performed by TRLFS, where EF4 was titrated with Eu^3+^. The deconvolution of TRLFS data using parallel factor analysis (PARAFAC) [44] are shown in Figure 2. The characteristics of the free Eu^3+^ aquo ion (green) can be clearly identified, where the ^5^D_0_ → ^7^F_1_ peak intensity (~590 nm) is higher than the hypersensitive ^5^D_0_ → ^7^F_2_ peak (~620 nm). The increase in the intensity of the hypersensitive peak is a sign of complex formation as can be seen from the spectrum (magenta, Figure 2). The hypersensitive ^5^D_0_ → ^7^F_2_ transition is denoted as such due to its strong sensitivity to the chemical environment of Eu^3+^. According to Judd–Ofelt theory, its intensity is strongly suppressed in centrosymmetric coordination environments and increases upon symmetry reduction induced by ligand binding [45]. The observed intensity increase therefore serves as a direct spectroscopic proof of Eu^3+^ complex formation. The decay time of Eu^3+^ aquo ion is 111.6 ± 1.0 µs and is in a good agreement with the literature value (110 ± 10 µs) [46, 47]. The decay time of a second component that we attribute to the 1:1 Eu^3+^‐EF4 complex is 165 ± 3.8 µs. The decay time of the Eu^3+^‐EF4 complex is longer than that of the Eu^3+^ aquoion due to less quenching by water molecules. The Eu^3+^ aquo ion is surrounded by 8–9 water molecules in its first coordination sphere, and its short decay time is due to energy transfer to O‐H oscillations of water molecules [48]. With complexation, the water molecules are replaced by EF4 donor groups and, as a consequence, reduced quenching of Eu^3+^ leads to increased decay times.

To get more insight into the formed complex species, a reverse titration scheme was performed with TRLFS as well (like in ITC experiments, Figure S2). Here, the EF4 was titrated with Eu^3+^. PARAFAC analysis (Figure S5) yielded again one complex with a decay time of ~160 µs, which we attributed to the 1:1 Eu^3+^‐EF4 complex. None of the titrations carried out in TRLFS experiments showed a distinct appearance of a 2:1 complex like in the ITC experiments. With an EF4 concentration of 10 µM and a very high metal concentration of more than 100 µM, the dataset was evaluated using a model comprising three europium species: the Eu^3+^ aquo ion, the 1:1 Eu^3+^‐EF4 complex, and the 2:1 Eu^3+^‐EF4 complex. The latter has been reported in the literature for other peptides [49, 50]. As shown in Figure S5, although it is possible to deconvolute the dataset using this more complex model, the emission spectra as well as the luminescence decay profiles of the complexes exhibit a high degree of self‐similarity. Therefore, for all subsequent TRLFS experiments, an experimental setup was chosen in which the concentrations were sufficiently low (1 µM) such that only the formation of the 1:1 Eu^3+^‐EF4 complex is expected.

PARAFAC results for complexation of other Ln (Tb^3+^, Sm^3+^) with EF4 similarly only generated two Ln^3+^ species, the aquo ion and the 1:1 Ln^3+^‐EF4 complex (Figures S6 and S7). Attempts to find indications of a 2:1 Ln^3+^‐EF4 complex were unsuccessful due to insignificant changes in the luminescence spectra of Tb^3+^ and Sm^3+^, as in the case of Eu^3+^. Determination of the number of species for Tb^3+^ and Sm^3+^ complexes can only rely on the decay time information.

Dissociation constants were calculated from the ITC and TRLFS data. Table 1 gives an overview of 1:1 and 2:1 complexes with the investigated Ln ions. The *K*_D_ values are in a similar lower micromolar range of 6.7–9.5 µM in ITC measurements and in the range of 0.8–1.7 µM in TRLFS. Although being in the same order of magnitude, the determined constants are not identical. We assume that this is caused by slightly different experimental conditions. TRLFS experiments were conducted in acidic water (pH ~5) to avoid formation of unwanted Ln^3+^‐solvent/buffer complexes, which have been reported for Eu^3+^ and several buffers [51]. In contrast, ITC measurements were carried out in 100 mM KCl (pH 6.0). For the 2:1 Ln^3+^‐EF4 complexes, the *K*_D_ values differ more along the three Ln^3+^ ions tested and are significantly higher than for the corresponding 1:1 complexes, indicating a much lower affinity for the 2:1 complex formation. Affinities of calmodulin’s EF‐hands toward Sm^3+^, Eu^3+^ and Tb^3+^ are reported in the literature [43, 52, 53, 54]. In most cases, though, *K*_D_ values have been determined for protein constructs but seldom for short peptides. Embedded in or fused to a protein, respectively, similar affinities for Ln^3+^ as in the native CaM were determined. In native CaM, the dissociation constant for Eu^3+^ varies across the four EF‐hands and is 60 nM for EF4, used in this study as a peptide [43]. One study reported a *K*_D_ value for the Tb^3+^‐EF4 peptide complex to be 72 ± 10 µM. However, in this study the EF4 was grafted to a small protein that functioned as a backbone, and the experiment was conducted using buffer and adjusted ionic strength at physiological pH [52]. Other studies reported *K*_D_ for Tb^3+^ of peptides based on EF‐hand motif but with variable peptide length [53, 54]. The *K*_D_ values were in the 10 µM range for all peptides and measured in buffer.

**TABLE 1:** Summary and comparison of the *K*_D_ values (in µM) for different Ln ions obtained by Ln^3+^ EF4 titration experiments using ITC and TRLFS. The difference in *K*_D_ values from ITC and TRLFS measurements is assumed to be solely caused by different setups, concentration ranges, solvents, and experimental errors.

	***K***_**D**_ ***,*** **µM, measured by**		
	ITC	TRLFS	ITC
Ln^3+^‐EF4 complex^a^	1:1		2:1
Sm^3+^	6.9 ± 0.3	0.85 ± 0.12	115 ± 4.5
Eu^3+^	6.8 ± 0.4	1.7 ± 0.1	99 ± 11
Tb^3+^	9.5 ± 0.6	1.4 ± 0.1	84 ± 5

a Stoichiometries (1:1, 2:1) were fixed for all speciation calculations during the fitting procedure. For the ITC titrations, a correction factor for the peptide concentration (n) was included as an additional fitted parameter (see Table S1); across all experiments and models, n ranged from 0.8–1.2, which is within the acceptable range for the assumed stoichiometry. For TRLFS, the parameter n could not be reliably fitted, as it strongly correlates with the parameter for differences in quantum yield of the different Eu^3+^ environments.

A reason why 2:1 Ln^3+^‐EF4 complexes were not observed in TRLFS experiments might be that the photophysical properties of the 2:1 complex are too similar to one of the other species (aquo ion or 1:1 complex). Moreover, since the complex is best seen at a large excess of Eu^3+^, the aquo ion dominates the overall signal. This makes a reliable separation of the spectral components challenging, and quantification is also difficult because the quantum efficiency of the aquo ion is low, leading to an overestimation of the complexes. The results of a PARAFAC analysis assuming the formation of a 1:1 and a 2:1 complex are shown in the (Figure S5). The strong resemblance of the kinetics as well as the spectrum of the 2:1 complex to the other species (together with the unknown quantum efficiencies of the species) showed that the resolving power of PARAFAC based on the available data set in the present case is not strong enough to yield trustworthy results for a 2:1 complex. The problem of temporary and small quantity complexes, and thus the correct choice of complex model, has already been discussed by Gutenthaler‐Tietze et al. [49, 50].

After analyzing the thermodynamics of the Ln‐peptide binding process, we next looked into structural characteristics of the complexes. NMR spectroscopy and MD simulations were the methods of choice to explore the binding sites, to further investigate possible stoichiometries, and to determine the dynamics of the peptide in solution.

### 3.2 Structural Characterization: Determination of Binding Sites

For a detailed analysis of the binding of EF4 to Ln with NMR spectroscopy, a complete assignment of signals of the peptide was established (Table S2). A set of NMR experiments was acquired to analyze the structural changes in EF4 upon its binding with Ln. We started by performing a pH titration of the free ^15^N,^13^C‐labeled peptide. ^15^N/^13^C/^1^H chemical shifts showed a strong dependence on pH, reflecting the protonation‐deprotonation processes of carboxylic groups in the peptide. As a result, it was possible to determine pKa values of these processes [55]. From the obtained data, it was found that for aspartic acid residues, the pKa value for the D1 residue is very different from the values for D3 and D5, which is because this residue is N‐terminal (Table S3). For residues D3 and D5, the pKa values are very similar, which indicates a high similarity between them in the free peptide. The glutamic acid residues in the peptide have the same pKa values. Also, the pKa values for all amino acids except the first one are slightly higher than the theoretical values. Since the pKa values are one unit or more below the pH value of 5.2 adjusted in the samples used for Ln binding studies, in these samples, all side chain carboxyl groups are at least 90% in the deprotonated state.

**FIGURE 3:**
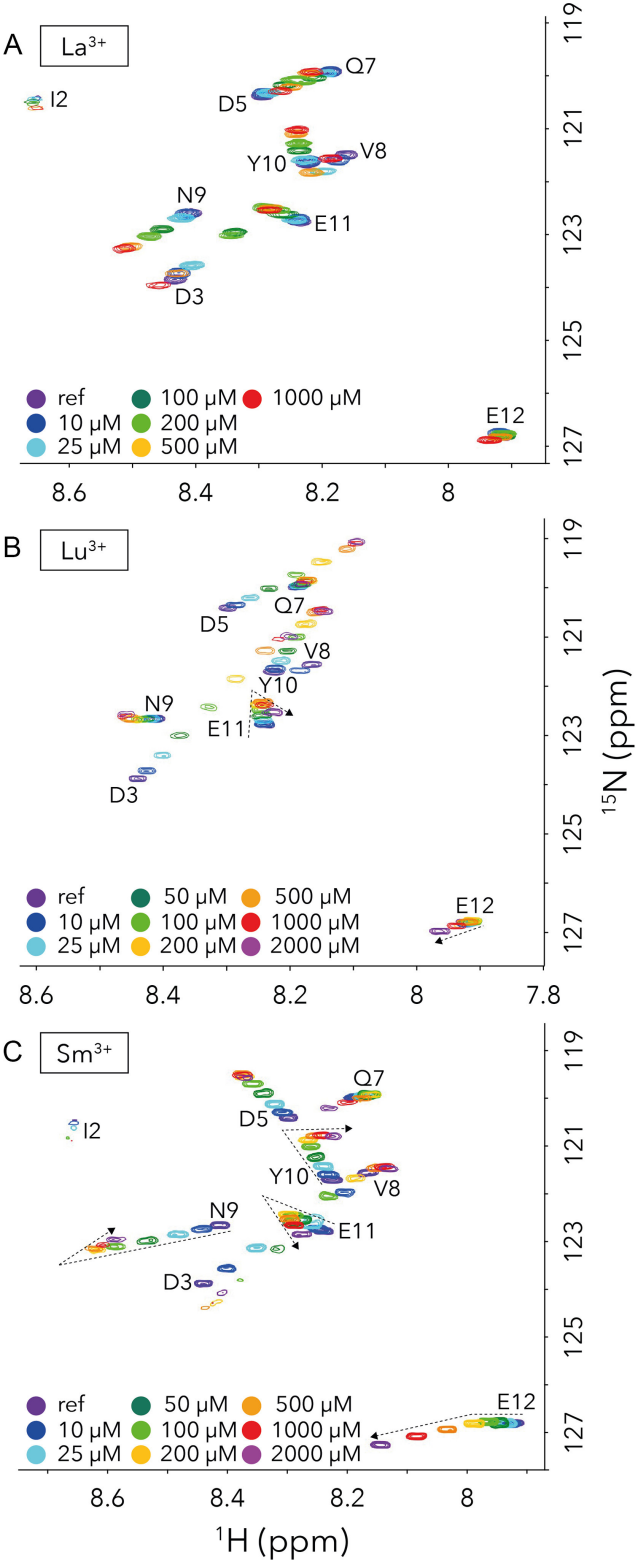
NMR titration of EF4 with Ln^3+^. Parts of ^1^H‐^15^N‐HSQC spectra show chemical shift changes upon titration of 100 µM ^15^N‐labeled EF4 with La^3+^ (A), Lu^3+^ (B), and Sm^3+^ (C) at pH 5.2. Experimental conditions and details on data acquisition and analysis are provided in the Supporting Information.

In the next step, ^15^N‐labeled EF4 titration was carried out with La^3+^, Lu^3+^, and Sm^3+^ (Figure 3). For other paramagnetic Ln, the strong paramagnetic relaxation led to the near‐complete disappearance of most signals in the spectra. For Eu^3+^ and Ce^3+^, which follow right after Sm^3+^ in the series of increasing paramagnetic relaxation enhancement (PRE), most of the peaks disappeared at a concentration of only 50 µM Ln^3+^ (Figure S8) [56]. For ^1^H‐^15^N‐HSQC spectra with La^3+^, Lu^3+^ and Sm^3+^, all peaks could be detected and assigned. In all cases, the concentration dependences of the chemical shift perturbations (CSPs) are indicative of the complexes being in fast exchange with the free states on the chemical shift timescale [57]. As can be seen from the titration (Figure 3), CSPs for the residue E12 in the presence of La^3+^ and Lu^3+^ were only observed at concentrations higher than 1000 µM of Ln^3+^. This could be an indication of EF4 binding to a second Ln^3+^ cation by forming a 2:1 Ln^3+^‐EF4 complex. Noteworthy, titration spectra with Sm^3+^ revealed a change in the nature of the CSPs for the residues of the C‐terminal part of the peptide from N9 at concentrations of Sm^3+^ around 200 µM and above, whereas the N‐terminal fragment of the peptide chain (residues I2‐V8) did not show these changes. This confirms the conclusion that 2:1 Ln^3+^‐EF4 complexes were formed at high concentrations of Ln^3+^ ions (≥200 µM for Sm^3+^ and ≥1000 µM for La^3+^ and Lu^3+^). This observation reflects higher binding affinity for Sm^3+^ in comparison to La^3+^ and Lu^3+^ for both the 1:1 and the 2:1 complex. It is worth noting that the last residue of the peptide does not participate in the formation of the 1:1 complex at lower concentrations of the Ln^3+^ ions. However, in the context of the CaM protein, this residue participates directly in metal coordination and is embedded in a structurally rigid α‐helical segment [58]. The first Ln‐binding site in the EF4 peptide is located in the N‐terminal half, as evidenced by rather strong CSPs at low Ln^3+^ concentrations during the titration, and weaker CSPs upon approaching saturation. The second Ln^3+^ binds to the C‐terminal half of the peptide, as evidenced by the change in the behavior of CSPs. It was found that the amide signal originating from residue D5 disappears already at very low concentrations of Eu^3+^ and Ce^3+^, which further supports the localization of the high‐affinity binding site near this residue. However, it is very difficult to identify which specific amino acids are involved in coordination, based barely on NMR spectroscopy.

**FIGURE 4:**
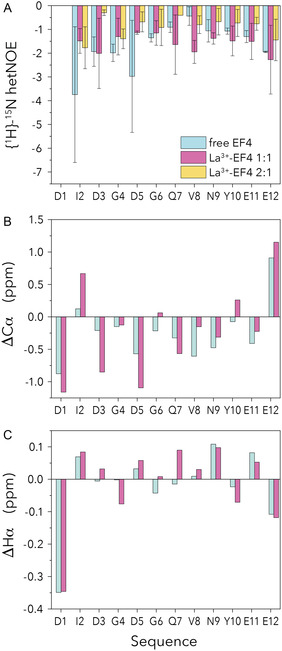
^1^H‐^15^N heteronuclear NOE of free EF4 and La^3+^ complexes (A). Deviations of ^13^Cα (B) and Hα (C) chemical shifts from random‐coil values measured at a magnetic field of 600 MHz and a temperature of 25 °C. Concentrations of samples were next: for free EF4 *c* = 1 mM, for 1:1 La^3+^‐EF4 complex c_La3+_ = 0.9 mM, c_EF4_ = 0.85 mM, for 2:1 La^3+^‐EF4 complex c_La3+_ = 10 mM, c_EF4_ = 0.2 mM. Experimental conditions and details on data acquisition and analysis are provided in the Supporting Information.

To gain insight into the conformation and conformational dynamics of the free EF4 peptide and the La^3+^‐EF4 complex, we performed a detailed characterization by further NMR experiments. First, NOESY NMR spectra were acquired (Figure S9). For both structures, only short‐range and medium‐range NOE‐based distance restraints between neighboring residues were found. This indicates a high flexibility of both the free peptide and the La^3+^‐EF4 complex. It is also worth noting that fairly intense NOE signals were found between the tyrosine (Y10) and valine (V8) residues for the La^3+^‐EF4 complex. These are structurally relevant and indicate that the side chains of these two residues are close to each other over longer periods of time. Since no long‐range NOE were found during the assignment of NOESY spectra, additional studies of the dynamics by {^1^H}‐^15^N heteronuclear NOE (hetNOE) experiments were carried out. As shown in Figure 4A, all values of the hetNOE spectra are negative, indicating high flexibility of both the free EF4 and the La^3+^‐EF4 complex. As rigid systems typically exhibit hetNOE values between 0.5 and 1, this indicates that even in the presence of La^3+^, the EF4 peptide does not adopt a well‐defined 3D structure. Rather, it dynamically interchanges between a multitude of conformations. Most likely, this includes varying binding modes and partially bound structures. Nevertheless, the hetNOE data show that the free peptide exhibits larger negative values in the N‐terminal half. Interestingly, at higher concentration of La^3+^, allowing for the formation of a 2:1 complex, the hetNOE values in the C‐terminal half of the peptide are somewhat smaller than in the free and 1:1 states. This is further evidence that, at high concentration of Ln^3+^, additional interactions lead to peptide structures with two Ln^3+^ ions bound, at least temporarily.

The deviation of ^13^Cα and Hα chemical shifts from random‐coil values is a good indicator of polypeptide secondary structure (Figure 4B,C) [59]. In line with our results from hetNOE measurements, for the La^3+^‐EF4 complex, a greater deviation compared to the free peptide was observed, indicating that the complex becomes the free peptide do not differ from random coil by more than 1 ppm for Cα and 0.5 ppm for Hα. Consequently, structure calculations based on NOEs resulted in heterogeneous structural ensembles, and even when including RDCs, structure calculations did not yield defined conformations of the La^3+^‐EF4 complex (data not shown).

Since the precise identification of binding sites remains challenging, MD simulations were performed to provide a more detailed picture of the complex formation and dynamic interactions.

### 3.3 Dynamics of the La^3+^‐EF4 Complex

The ITC, TRLFS, and NMR investigations revealed the formation of 1:1 and 2:1 complexes between several Ln^3+^ and EF4, but the binding sites are difficult to assign. Therefore, MD simulations were performed with special emphasis on the coordinating amino acids. As both 1:1 and 2:1 complexes were found experimentally, simulations with those conditions were started for La^3+^, Sm^3+^, and Eu^3+^ over a time period of 100 ns. A detailed overview of the starting conditions for every MD simulation is given in the Supporting Information (Table S4).

**FIGURE 5:**
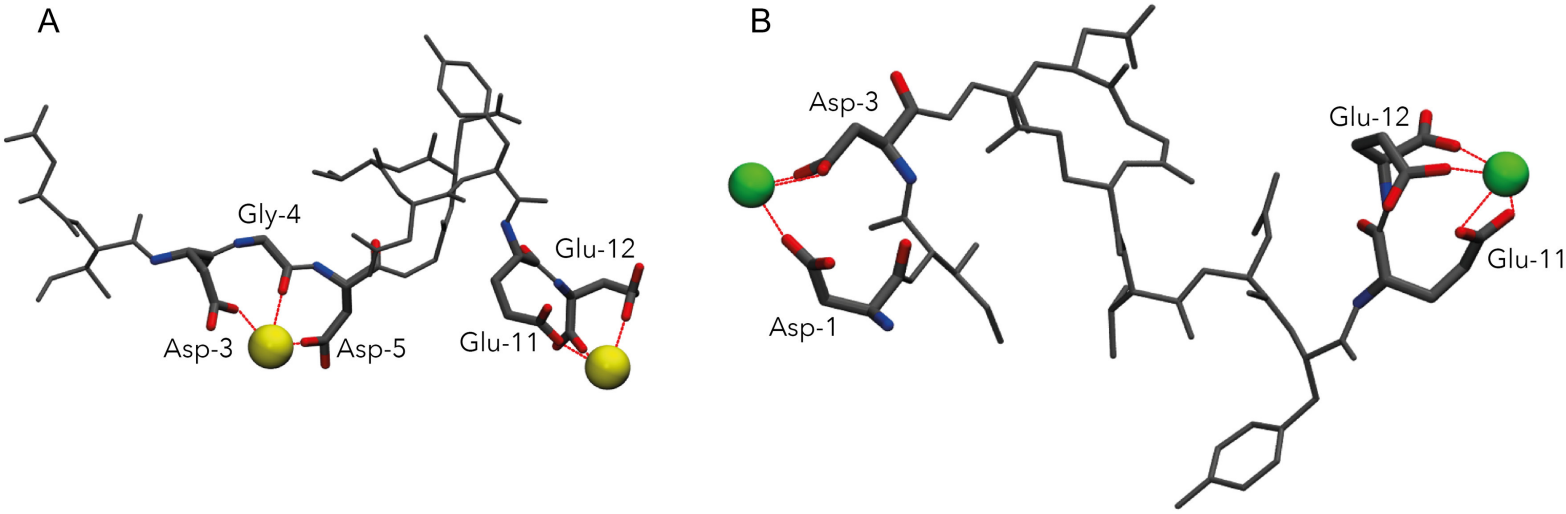
Examples for simulated structures of Ln_2_‐EF4 complexes with Sm^3+^ (A) and Eu^3+^ (B).

In good agreement with the NMR data, the MD simulations show that the Eu^3+^‐EF4 system is highly dynamic. The MD simulations were dominated by two distinct binding motifs from a peptide perspective. Motif 1 involves carboxylate coordination through D1, D3, and D5. In Motif 2, E11, E12, and the carboxyl group of the C‐terminus are involved in the metal coordination. These two motifs can be combined into a metal–peptide complex with higher stoichiometry (2:1 Ln^3+^‐EF4 complex), as exemplified in Figure 5. The average coordination numbers for the MD simulations are listed in Tables S5–S7. Due to the dynamics in the simulated systems, the number of coordinating amino acids fluctuates, as does the denticity of the carboxylates. Consistently, 5–7 water molecules remain in the first coordination sphere of the metal. Therefore, a definitive structure for the complexes cannot be extracted from the simulation. The high dynamics observed in the systems were found for all three investigated Ln^3+^ ions, overshadowing any minor differences that might exist.

To summarize the results of NMR spectroscopic studies and MD simulations, these techniques suggest that the Ln^3+^‐peptide complexes neither have defined coordination patterns nor rigid structures in solution. Although the Ln^3+^‐peptide complex is more rigid than the free peptide, it was not possible to calculate well‐defined structures. In addition, various complexes can be formed under suitable conditions, demonstrating the flexibility of the Ln^3+^‐peptide system.

Comparing EF4 with other lanthanide‐binding peptides reveals both shared coordination principles and distinctive structural features. In canonical EF‐hand loops, metal coordination follows the X, Y, Z,−Y,−X,−Z scheme, where positions 1(X), 3(Y), 5(Z), 9(−X), and 12(−Z, bidentate glutamate) provide oxygen donors. EF4 carries aspartate or glutamate at all canonical coordination positions, consistent with our MD‐derived binding motifs (D1/D3/D5 as primary site, E11/E12 as secondary site). A striking contrast to EF4 arises at position 2: whereas EF4 carries isoleucine at this noncoordinating position, LanM bears a proline in all four of its EF‐hand loops. This substitution introduces backbone rigidity and constrains the loop geometry in a manner that is particularly complementary to the ionic radius of early Ln, contributing to LanM’s remarkable picomolar affinity and selectivity for light Ln over Ca^2+^. In the isolated peptide context, however, the structural role of proline shifts rather than simply abolishes binding: as shown by Gutenthaler et al. [49, 50], the proline‐induced backbone geometry causes the N‐terminal aspartate to point away from the binding site in the detached LanM EF‐hand loops, reducing affinity and displacing the primary coordination motif toward the C‐terminus. This contrasts with calmodulin’s EF4, where the N‐terminal aspartate residues D1, D3, and D5 form the dominant coordination center in the 1:1 complex. The engineered LBT achieves high affinity through a distinct strategy: aromatic stacking interactions involving tryptophan and tyrosine residues stabilize a defined turn conformation, effectively mimicking the rigidity otherwise provided by the protein scaffold, thereby achieving nanomolar affinity despite its short peptide length.

### 3.4 Peptide Immobilization and Extraction of Sm^3+^

Our results described so far indicate that the free peptide EF4 behaves very differently in solution than the corresponding part of the protein CaM. The peptide exhibits much higher dynamics without adopting a specific structure upon binding to the Ln^3+^ cations. However, despite the less defined structures compared to the Ln‐protein complexes, high affinities for Ln binding are preserved. Therefore, the free peptide represents an interesting system for the recovery of Ln ions. For industrial applications, a reliable immobilization of peptides while maintaining their functionality is necessary. We have previously shown that the polymer conjugation to EF4 has a distinct influence on the binding affinity to Eu^3+^, probably by restricting the dynamics [60]. Notably, for the polymer‐conjugated EF4 only a 1:1 stoichiometry was observed, suggesting that immobilization suppresses the secondary 2:1 binding motif – an effect that is likely to apply to the resin‐bound peptide as well.

We chose to assemble the peptide through fluorenylmethoxycarbonyl (Fmoc) solid‐phase peptide synthesis [61] directly on a suitable matrix. This comes with the advantage that biomolecules can be grafted with high densities, without the need for prior purification and an additional binding step to the matrix, which can be inefficient. The EF4 peptide was synthesized on polystyrene‐based TentaGel microbeads with a base‐labile 4‐(hydroxymethyl)benzoic acid (HMBA) linker [62]. Similar experiments were conducted simultaneously by Sree et al. (without mutual knowledge) [63]. The TentaGel matrix offers a hydrophilic environment for the peptide while also enabling high densities of metal‐binding ligands.

Many standard SPPS matrices like Wang‐ or Rink‐Amide‐resin use acid‐labile linkers where cleavage of the peptide and side‐chain deprotection happen simultaneously [64]. The here‐used HMBA linker is resistant to acidic conditions, allowing the removal of the protection groups of the peptide side chains using trifluoroacetic acid, without cleaving the biomolecules themselves from the matrix [62]. Cleavage of the HMBA linker and consequently removal of the peptide from the resin can be conducted under alkaline conditions with various nucleophiles [65], thus allowing characterization of the SPPS product. Similar systems are well established in particle‐based one‐bead‐one‐peptide (OBOP) libraries [66, 67].

After removal of side chain protection groups, the complete synthesis of EF4 was validated by cleavage of the peptide from the resin using sodium hydroxide and subsequent analysis via high‐performance liquid chromatography (HPLC) and electrospray ionization mass spectrometry (ESI‐MS) (Figure S11), confirming good purity (Figure S12). The grafting density of the generated material was analyzed through cleavage of the residual N‐terminal Fmoc‐protection group and subsequent UV−vis quantification of the resulting dibenzofulvene‐adduct. The density of the immobilized peptide was determined to be 177.7 µmol/g.

Long‐term stability must be ensured for an efficient application, especially as the binding of Ln^3+^ is conducted under slightly acidic conditions of pH 6. To evaluate the stability in an aqueous environment, the EF4‐functionalized resin with an Fmoc‐protected N‐terminus was incubated for 5 days at pH 2 (10 mM HCl), pH 5 (100 mM acetate buffer), and pH 7.4 (100 mM phosphate buffer) or 1 day in aq. 1 M HNO_3_. Peptide loading was then deter‐mined by Fmoc cleavage. For all pH ranges, no change in peptide loading was determined (Table S8), thus indicating sufficient stability.

**FIGURE 6:**
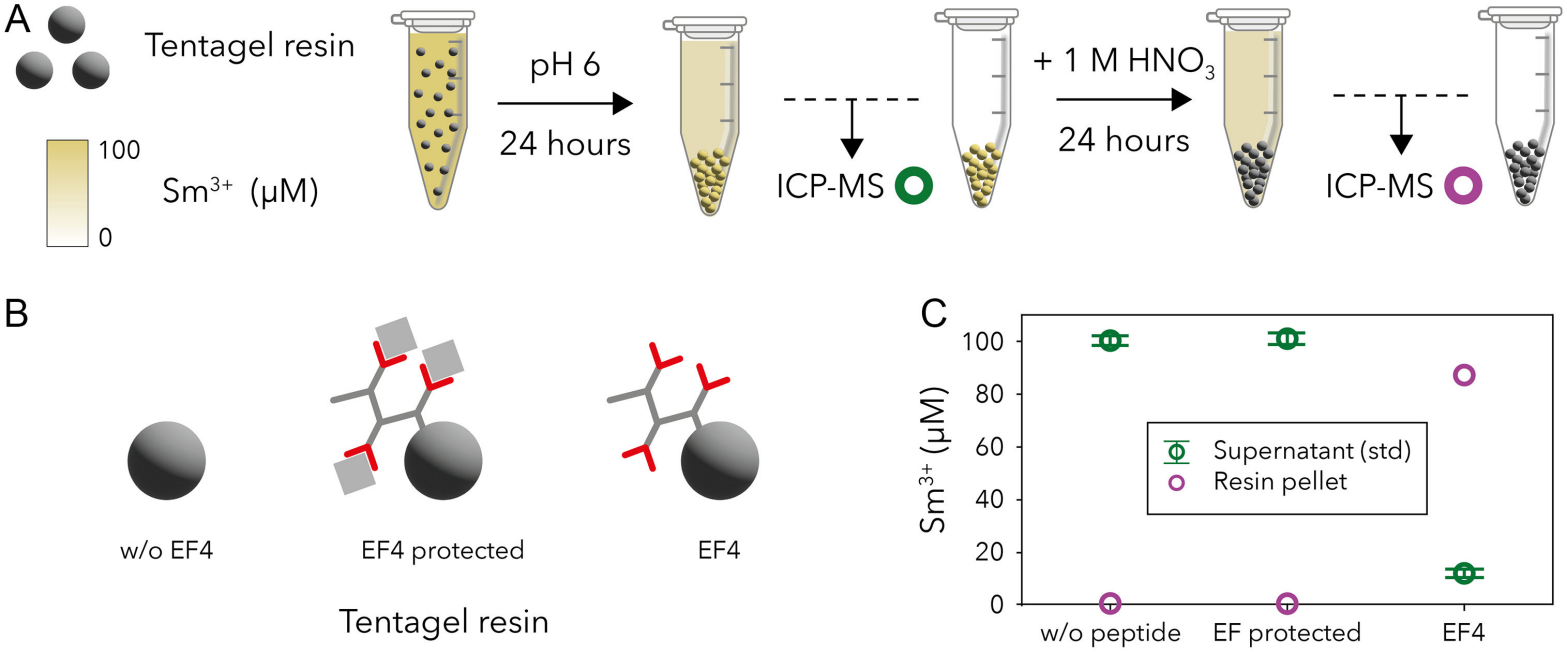
Extraction of samarium ions with immobilized EF4 peptide (A). Functionalized resin was incubated with Sm^3+^ for 24 h at pH 6. After centrifugation, the supernatant with significantly reduced metal ion concentration was removed and analyzed by ICP‐MS (C). Resin‐bound Sm^3+^ was recovered by treatment with 1 M HNO_3_. Samarium concentration is visualized by a yellow color tint (Image was created with BioRender). Samarium binding to different resin species (B) was analyzed by ICP‐MS (C).

The capability of the functionalized resin to bind Ln^3+^ ions was tested next. Sm^3+^ was chosen as a model system because of its high affinity to the EF4 peptide. The resin‐bound peptide was incubated with equimolar amounts of Sm^3+^ (100 µM EF4 :100 µM SmCl_3_) at pH 6. Nonfunctionalized resin and resin‐bound EF4 with side chain protection served as reference systems. Suspensions were shaken for 24 h at room temperature, centrifuged, and the supernatant was analyzed for metal ion concentration via inductively coupled plasma‐mass spectrometry (ICP‐MS). While plain TentaGel and side‐chain‐protected EF4‐resin showed no binding of Sm^3+^, the deprotected EF4 peptide bound 88% of the available samarium ions, corresponding to an adsorption capacity of 156.4 µmol Sm^3+^ per g resin (Figure 6). Adding to that, it was possible to recover the metal ions from the peptides almost quantitatively (99%) by treatment of the resin with 1 M HNO_3_ (Figure 6).

Chemical microscopy was previously shown to be a powerful tool for spatial resolved Eu^3+^ speciation studies [68, 69]. Instead of Sm^3+^, Eu^3+^ had to be used since Sm^3+^ does not absorb the wavelength of the laser. The binding of Eu^3+^ to the resin was confirmed by chemical microscopy through excitation at 532 nm. The emission spectrum of Eu^3+^ and the microscopy image of the resin with bound Eu^3+^ are shown in the Supporting Information (Figure S13). A clear co‐localization of the particle and Eu^3+^ was observed, suggesting a homogeneous binding of the Ln^3+^ ions to the solid support.

## 4 Conclusion

We explored thermodynamic and structural properties of the EF4 peptide in solution when complexed with selected Ln^3+^ ions. In general, high affinities were found for the Ln^3+^‐EF4 complexes. We determined *K*_D_ values around 1 µM by TRLFS and around 10 µM by ITC, which are in good agreement with previous reports on other EF4 constructs. The isolated EF4 peptide exhibits approximately 30‐fold (vs. TRLFS) to 110‐fold (vs. ITC) weaker affinities to Ln^3+^ ions than in native calmodulin’s EF‐hand 4.

NMR spectroscopy and MD simulations revealed a high flexibility of the peptide in solution, both for the free peptide and the Ln^3+^‐EF4 complexes. Therefore, no uniquely defined structure could be determined. In comparison to the peptide sequence in CaM, the free peptide may form a variety of different yet experimentally indistinguishable complexes with Ln^3+^ ions. While a 1:1 stoichiometry was most abundant, ITC and NMR analysis also revealed evidence for the binding of two Ln^3+^ ions to one peptide at high Ln^3+^ concentrations and in excess with respect to the peptide. According to the MD simulations and corroborated by hetNOE measurements, two classes of binding motifs for Ln^3+^ ions are present in the peptide. The primary binding motif consists of D1, D3, and D5 residues, forming the main coordination center observed in the 1:1 complex. The secondary binding site, involving E11 and E12, has significantly weaker affinity and only becomes relevant at high Ln^3+^‐to‐peptide ratios. This could be a reason why the 2:1 Ln^3+^‐EF4 was not detected by TRLFS.

To demonstrate the extraction of Ln^3+^ from an aqueous solution, EF4 was immobilized on micrometer‐particles. Sm^3+^ was effectively bound to the solid phase and could be recovered from the peptide complexes almost quantitatively by washing with acidic solution.

With this study, we provide a detailed biophysical characterization of Ln^3+^ binding to the isolated EF4 peptide, bringing together multiple perspectives. The results deepen our understanding of how peptides recognize Ln^3+^ ions and may trigger future research to develop bio‐inspired Ln^3+^ ligands with improved affinity and selectivity.

Furthermore, our study demonstrates that EF4 is a promising tool for the separation and concentration of Ln^3+^ from Ln^3+^‐containing water bodies. Next research efforts on the way to the application of peptides as efficient separation tools for the various Ln will be the engineering of peptide sequences, optimization of the immobilization matrix, and investigation of the separation of multiple Ln^3+^ ions from solution. Techniques such as phage surface display and computer‐based peptide prediction may help to identify artificial peptides with specific binding characteristics for individual Ln. Affinity and selectivity could be further improved by rigidification, e.g., through head‐to‐tail cyclization, as recently demonstrated for LanM‐derived peptides where cyclization increased lanthanide affinity and reduced the number of formed complex species [21]. Moreover, the influence of the type and length of conjugated polymers on the binding affinity to various Ln will be investigated. In the long run, our research may contribute to the development of a modular hybrid Ln filter and the installation of sustainable Ln recycling technologies. Therefore, our study demonstrates the potential of bioinspired approaches to develop a separation platform for Ln recovery from wastewater and its simultaneous bioremediation.

## Funding

This study was supported by the Federal Ministry of Research, Technology and Space (031B1122) and Deutsche Forschungsgemeinschaft (530253155).

## Conflicts of Interest

The authors declare no conflicts of interest.

## Supporting information

Detailed experimental procedures, characterization data, and additional figures are provided in the Supporting Information. The authors have cited additional references within the Supporting Information [70, 71, 72, 73, 74].

## Data Availability

The data that support the findings of this study are openly available in Rodare at https://doi.org/10.14278/rodare.4284, reference number 10.14278/rodare.4284.

## References

[1] Y. Geng , J. Sarkis , and R. Bleischwitz , “How to Build a Circular Economy for Rare‐Earth Elements,” Nature 619 (2023): 248–251. 37430110 10.1038/d41586-023-02153-z

[2] K. Binnemans , P. T. Jones , T. Müller , and L. Yurramendi , “Rare Earths and the Balance Problem: How to Deal with Changing Markets?,” Journal of Sustainable Metallurgy 4 (2018): 126–146.

[3] P. Cen , X. Bian , Z. Liu , M. Gu , W. Wu , and B. Li , “Extraction of Rare Earths from Bastnaesite Concentrates: A Critical Review and Perspective for the Future,” Minerals Engineering 171 (2021): 107081.

[4] M. Grohol and C. Veeh , Study on the Critical Raw Materials for the EU. 2023 ‐ Final Report (Publications Office Of The European Union, 2023).

[5] H. Wang , S. Zhang , B. Li , D. Pan , Y. Wu , and T. Zuo , “Recovery of Waste Printed Circuit Boards through Pyrometallurgical Processing: A Review,” Resources, Conservation, and Recycling 126 (2017): 209–218.

[6] R. K. Jyothi , T. Thenepalli , J. W. Ahn , P. K. Parhi , K. W. Chung , and J.‐Y. Lee , “Review of Rare Earth Elements Recovery from Secondary Resources for Clean Energy Technologies: Grand Opportunities to Create Wealth from Waste,” Journal of Cleaner Production 267 (2020): 122048.

[7] C. Ramprasad , W. Gwenzi , N. Chaukura , et al., “Strategies and Options for the Sustainable Recovery of Rare Earth Elements from Electrical and Electronic Waste,” Chemical Engineering Journal 442 (2022): 135992.

[8] R. P. Oliveira , J. Benvenuti , and D. C. R. Espinosa , “A Review of the Current Progress in Recycling Technologies for Gallium and Rare Earth Elements from Light‐Emitting Diodes,” Renewable and Sustainable Energy Reviews 145 (2021): 111090.

[9] L. Castro , M. L. Blázquez , F. González , and J.Á. Muñoz , “Biohydrometallurgy for Rare Earth Elements Recovery from Industrial Wastes,” Molecules 26 (2021): 6200. 34684778 10.3390/molecules26206200 PMC8538766

[10] K. Binnemans , P. T. Jones , B. Blanpain , et al., “Recycling of Rare Earths: A Critical Review,” Journal of Cleaner Production 51 (2013): 1–22.

[11] D. Kim , L. E. Powell , L. H. Delmau , E. S. Peterson , J. Herchenroeder , and R. R. Bhave , “Selective Extraction of Rare Earth Elements from Permanent Magnet Scraps with Membrane Solvent Extraction,” Environmental Science & Technology 49 (2015): 9452–9459. 26107531 10.1021/acs.est.5b01306

[12] S. Demir , N. K. Brune , J. F. Van Humbeck , et al., “Extraction of Lanthanide and Actinide Ions from Aqueous Mixtures Using a Carboxylic Acid‐Functionalized Porous Aromatic Framework,” ACS Central Science 2 (2016): 253–265. 27163056 10.1021/acscentsci.6b00066 PMC4850516

[13] X. Zhao , M. Wong , C. Mao , et al., “Size‐Selective Crystallization of Homochiral Camphorate Metal–Organic Frameworks for Lanthanide Separation,” Journal of the American Chemical Society 136 (2014): 12572–12575. 25164942 10.1021/ja5067306

[14] R. Paz , N. K. Gupta , H. Viltres , et al., “Lanthanides Adsorption on Metal‐Organic Framework: Experimental Insight and Spectroscopic Evidence,” Separation and Purification Technology 298 (2022): 121606.

[15] Y. Hosokawa , A. Oshima , T. Hatanaka , and N. Ishida , “Improved Recovery and Selectivity of Lanthanide‐Ion‐Binding Cyclic Peptide Hosts by Changing the Position of Acidic Amino Acids,” Minerals 12 (2022): 148.

[16] T. Hatanaka , A. Matsugami , T. Nonaka , et al., “Rationally Designed Mineralization for Selective Recovery of the Rare Earth Elements,” Nature Communications 8 (2017): 15670. 10.1038/ncomms15670 PMC5458567 28548098

[17] L. E. Ortuno Macias , H. Zhang , B. M. Ocko , K. J. Stebe , C. Maldarelli , and R. S. Tu , “Enhanced Rare Earth Element Recovery with Cross‐Linked Glutaraldehyde‐Lanthanide Binding Peptides in Foam‐Based Separations,” Journal of Colloid and Interface Science 678 (2025): 1153–1164. 39270572 10.1016/j.jcis.2024.08.225

[18] F. L. Lederer , S. B. Curtis , S. Bachmann , W. S. Dunbar , and R. T. A. MacGillivray , “Identification of Lanthanum‐Specific Peptides for Future Recycling of Rare Earth Elements from Compact Fluorescent Lamps,” Biotechnology and Bioengineering 114 (2017): 1016–1024. 27987304 10.1002/bit.26240

[19] K. Pollmann , S. Kutschke , S. Matys , J. Raff , G. Hlawacek , and F. L. Lederer , “Bio‐Recycling of Metals: Recycling of Technical Products Using Biological Applications,” Biotechnology Advances 36 (2018): 1048–1062. 29555455 10.1016/j.biotechadv.2018.03.006

[20] F. L. Lederer , R. Braun , L. M. Schöne , and K. Pollmann , “Identification of Peptides as Alternative Recycling Tools via Phage Surface Display – How Biology Supports Geosciences,” Minerals Engineering 132 (2019): 245–250.

[21] S. M. Gutenthaler‐Tietze , J. Kretzschmar , S. Tsushima , et al., “Influence of Head‐to‐Tail Cyclisation of Lanmodulin‐Inspired Peptides on Lanthanide Affinity and Structure,” Chemistry – A European Journal (2026): e71438.42501354 10.1002/chem.71438

[22] R. Williams , “Calcium and Calmodulin,” Cell Calcium 13 (1992): 355–362. 1505001 10.1016/0143-4160(92)90049-x

[23] M. Ikura , “Calcium Binding and Conformational Response in EF‐Hand Proteins,” Trends in Biochemical Sciences 21 (1996): 14–17. 8848832

[24] D. Chin and A. R. Means , “Calmodulin: A Prototypical Calcium Sensor,” Trends in Cell Biology 10 (2000): 322–328. 10884684 10.1016/s0962-8924(00)01800-6

[25] L. A. Onek and R. J. Smith , “Calmodulin and Calcium Mediated Regulation in Prokaryotes,” Microbiology 138 (1992): 1039–1049. 10.1099/00221287-138-6-1039 1527487

[26] J. A. Cotruvo Jr. , E. R. Featherston , J. A. Mattocks , J. V. Ho , and T. N. Laremore , “Lanmodulin: A Highly Selective Lanthanide‐Binding Protein from a Lanthanide‐Utilizing Bacterium,” Journal of the American Chemical Society 140 (2018): 15056–15061. 30351021 10.1021/jacs.8b09842

[27] H. Singer , R. Steudtner , I. Sottorff , et al., “Learning from Nature: Recovery of Rare Earth Elements by the Extremophilic Bacterium Methylacidiphilum Fumariolicum,” Chemical Communications 59 (2023): 9066–9069. 37382581 10.1039/d3cc01341c PMC10358715

[28] J. L. Gifford , M. P. Walsh , and H. J. Vogel , “Structures and Metal‐Ion‐Binding Properties of the Ca^2+^‐Binding Helix–loop–helix EF‐Hand Motifs,” The Biochemical Journal 405 (2007): 199–221. 17590154 10.1042/BJ20070255

[29] I. Bertini , I. Gelis , N. Katsaros , C. Luchinat , and A. Provenzani , “Tuning the Affinity for Lanthanides of Calcium Binding Proteins,” Biochemistry 42 (2003): 8011–8021. 12834353 10.1021/bi034494z

[30] J. Hu , X. Jia , Q. Li , X. Yang , and K. Wang , “Binding of La^3+^ to Calmodulin and Its Effects on the Interaction between Calmodulin and Calmodulin Binding Peptide, Polistes Mastoparan,” Biochemistry 43 (2004): 2688–2698. 15005604 10.1021/bi035784i

[31] V. Nikolova , N. Kircheva , S. Dobrev , S. Angelova , and T. Dudev , “Lanthanides as Calcium Mimetic Species in Calcium‐Signaling/Buffering Proteins: The Effect of Lanthanide Type on the Ca^2+^/Ln^3+^ Competition,” International Journal of Molecular Sciences 24 (2023): 6297. 37047269 10.3390/ijms24076297 PMC10094714

[32] R. R. Biekofsky , F. W. Muskett , J. M. Schmidt , et al., “NMR Approaches for Monitoring Domain Orientations in Calcium‐Binding Proteins in Solution Using Partial Replacement of Ca^2+^ by Tb^3+^ ,” FEBS Letters 460 (1999): 519–526. 10556528 10.1016/s0014-5793(99)01410-6

[33] C. W. Hogue , J. P. MacManus , D. Banville , and A. G. Szabo , “Comparison of Terbium (III) Luminescence Enhancement in Mutants of EF Hand Calcium Binding Proteins,” The Journal of Biological Chemistry 267 (1992): 13340–13347. 1618836

[34] I. Bertini , C. Luchinat , G. Parigi , and R. Pierattelli , “NMR Spectroscopy of Paramagnetic Metalloproteins,” Chembiochem 6 (2005): 1536–1549. 16094696 10.1002/cbic.200500124

[35] G. Otting , “Prospects for Lanthanides in Structural Biology by NMR,” Journal of Biomolecular NMR 42 (2008): 1–9. 18688728 10.1007/s10858-008-9256-0

[36] G. Pintacuda , M. John , X.‐C. Su , and G. Otting , “NMR Structure Determination of Protein−Ligand Complexes by Lanthanide Labeling,” Accounts of Chemical Research 40 (2007): 206–212. 17370992 10.1021/ar050087z

[37] M. Nitz , M. Sherawat , K. J. Franz , E. Peisach , K. N. Allen , and B. Imperiali , “Structural Origin of the High Affinity of a Chemically Evolved Lanthanide‐Binding Peptide,” Angewandte Chemie International Edition 43 (2004): 3682–3685. 15248272 10.1002/anie.200460028

[38] K. Barthelmes , A. M. Reynolds , E. Peisach , et al., “Engineering Encodable Lanthanide‐Binding Tags into Loop Regions of Proteins,” Journal of the American Chemical Society 133 (2011): 808–819. 21182275 10.1021/ja104983t PMC3043167

[39] T. Hatanaka , N. Kikkawa , A. Matsugami , Y. Hosokawa , F. Hayashi , and N. Ishida , “The Origins of Binding Specificity of a Lanthanide Ion Binding Peptide,” Scientific Reports 10 (2020): 19468. 33173124 10.1038/s41598-020-76527-y PMC7656248

[40] K. J. Franz , M. Nitz , and B. Imperiali , “Lanthanide‐Binding Tags as Versatile Protein Coexpression Probes,” Chembiochem 4 (2003): 265–271. 12672105 10.1002/cbic.200390046

[41] M. Nitz , K. J. Franz , R. L. Maglathlin , and B. Imperiali , “A Powerful Combinatorial Screen to Identify High‐Affinity Terbium(III)‐Binding Peptides,” ChemBioChem 4 (2003): 272–276. 12672106 10.1002/cbic.200390047

[42] J. Wöhnert , K. J. Franz , M. Nitz , B. Imperiali , and H. Schwalbe , “Protein Alignment by a Coexpressed Lanthanide‐Binding Tag for the Measurement of Residual Dipolar Couplings,” Journal of the American Chemical Society 125 (2003): 13338–13339. 14583012 10.1021/ja036022d

[43] B. Drobot , M. Schmidt , Y. Mochizuki , et al., “Cm^3+^/Eu^3+^ Induced Structural, Mechanistic and Functional Implications for Calmodulin,” Physical Chemistry Chemical Physics 21 (2019): 21213–21222. 31418759 10.1039/c9cp03750k

[44] R. Bro , “PARAFAC. Tutorial and Applications,” Chemometrics and Intelligent Laboratory Systems 38 (1997): 149–171.

[45] K. Binnemans , “Interpretation of Europium(III) Spectra,” Coordination Chemistry Reviews 295 (2015): 1–45.

[46] T. Kimura and G. R. Choppin , “Luminescence Study on Determination of the Hydration Number of Cm(III),” Journal of Alloys and Compounds 213‐214 (1994): 313–317.

[47] J. C. G. Bünzli and J. R. Yersin , “Fluorescence Spectra and Lifetime Measurements of Aqueous Solutions of Europium Nitrate and Perchlorate,” Inorganic Chemistry 18 (1979): 605–607.

[48] B. Marmodée , K. Jahn , F. Ariese , C. Gooijer , and M. U. Kumke , “Direct Spectroscopic Evidence of 8‐ and 9‐Fold Coordinated Europium(III) Species in H_2_O and D_2_O,” The Journal of Physical Chemistry A 114 (2010): 13050–13054. 21121682 10.1021/jp1094036

[49] S. M. Gutenthaler , S. Tsushima , R. Steudtner , et al., “Lanmodulin Peptides – Unravelling the Binding of the EF‐Hand Loop Sequences Stripped from the Structural Corset,” Inorganic Chemistry Frontiers 9 (2022): 4009–4021. 36091973 10.1039/d2qi00933a PMC9362731

[50] S. M. Gutenthaler‐Tietze , J. Kretzschmar , S. Tsushima , R. Steudtner , B. Drobot , and L. J. Daumann , “Reversing Lanmodulin’s Metal‐Binding Sequence in Short Peptides Surprisingly Increases the Lanthanide Affinity,” Angewandte Chemie International Edition 64 (2025): e202510453. 40985823 10.1002/anie.202510453 PMC12603986

[51] P. Mandal , J. Kretzschmar , and B. Drobot , “Not Just a Background: PH Buffers Do Interact with Lanthanide Ions—a Europium(III) Case Study,” JBIC Journal of Biological Inorganic Chemistry 27 (2022): 249–260. 35150337 10.1007/s00775-022-01930-x PMC8907096

[52] Y. Ye , H.‐W. Lee , W. Yang , S. Shealy , and J. J. Yang , “Probing Site‐Specific Calmodulin Calcium and Lanthanide Affinity by Grafting,” Journal of the American Chemical Society 127 (2005): 3743–3750. 15771508 10.1021/ja042786x

[53] G. Borin , P. Ruzza , M. Rossi , A. Calderan , F. Marchiori , and E. Peggion , “Conformation and Ion Binding Properties of Peptides Related to Calcium Binding Domain III of Bovine Brain Calmodulin,” Biopolymers 28 (1989): 353–369. 2720113 10.1002/bip.360280134

[54] R. Buchta , E. Bondi , and M. Fridkin , “Peptides Related to the Calcium Binding Domains II and III of Calmodulin. Synthesis and Calmodulin‐Like Features,” International Journal of Peptide and Protein Research 28 (1986): 289–297. 2946646 10.1111/j.1399-3011.1986.tb03258.x

[55] M. A. S. Hass and F. A. A. Mulder , “Contemporary NMR Studies of Protein Electrostatics,” Annual Review of Biophysics 44 (2015): 53–75. 10.1146/annurev-biophys-083012-130351 25747592

[56] G. Otting , “Protein NMR Using Paramagnetic Ions,” Annual Review of Biophysics 39 (2010): 387–405. 10.1146/annurev.biophys.093008.131321 20462377

[57] M. P. Williamson , “Using Chemical Shift Perturbation to Characterise Ligand Binding,” Progress in Nuclear Magnetic Resonance Spectroscopy 73 (2013): 1–16. 23962882 10.1016/j.pnmrs.2013.02.001

[58] R. Chattopadhyaya , W. E. Meador , A. R. Means , and F. A. Quiocho , “Calmodulin Structure Refined at 1.7 Å Resolution,” Journal of Molecular Biology 228 (1992): 1177–1192. 1474585 10.1016/0022-2836(92)90324-d

[59] S. Spera and A. Bax , “Empirical Correlation between Protein Backbone Conformation and C.alpha. and C.beta. 13C Nuclear Magnetic Resonance Chemical Shifts,” Journal of the American Chemical Society 113 (1991): 5490–5492.

[60] D. Marlina , Y. Müllers , U. Glebe , and M. U. Kumke , “Spectroscopic Characterization of Europium Binding to a Calmodulin‐EF4 Hand Peptide–polymer Conjugate,” RSC Advances 14 (2024): 14091–14099. 38686292 10.1039/d4ra01505c PMC11056824

[61] G. B. Fields and R. L. Noble , “Solid Phase Peptide Synthesis Utilizing 9‐Fluorenylmethoxycarbonyl Amino Acids,” International Journal of Peptide and Protein Research 35 (1990): 161–214. 2191922 10.1111/j.1399-3011.1990.tb00939.x

[62] E. Atherton , C. J. Logan , and R. C. Sheppard , “Peptide Synthesis. Part 2. Procedures for Solid‐Phase Synthesis Using Nα‐Fluorenylmethoxycarbonylamino‐Acids on Polyamide Supports. Synthesis of Substance P and of Acyl Carrier Protein 65–74 Decapeptide,” Journal of the Chemical Society, Perkin Transactions 1 (1981): 538–546.

[63] H. Sree , G. Swarup , S. Gupta , and K. Pushpavanam , “Gravity‐Driven Separation for Enrichment of Rare Earth Elements Using Lanthanide Binding Peptide‐Immobilized Resin,” ACS Applied Bio Materials 7 (2024): 7828–7837. 10.1021/acsabm.3c01280 38685483

[64] S. L. Pedersen and K. J. Jensen , Peptide Synthesizer Application, eds. K.J. Jensen , P. Tofteng Shelton , S.L. Pedersen (Humana Press, 2013), 43–63.

[65] J. Hansen , F. Diness , and M. Meldal , “C‐Terminally Modified Peptides via Cleavage of the HMBA Linker by O ‐, N ‐ or S ‐Nucleophiles,” Organic & Biomolecular Chemistry 14 (2016): 3238–3245. 26924021 10.1039/c6ob00213g

[66] K. S. Lam , S. E. Salmon , E. M. Hersh , V. J. Hruby , W. M. Kazmierski , and R. J. Knapp , “A New Type of Synthetic Peptide Library for Identifying Ligand‐Binding Activity,” Nature 354 (1991): 82–84. 1944576 10.1038/354082a0

[67] J. O. Kaufmann , J. Brangsch , A. Kader , et al., “ADAMTS4‐Specific MR Probe to Assess Aortic Aneurysms In Vivo Using Synthetic Peptide Libraries,” Nature Communications 13 (2022): 2867. 10.1038/s41467-022-30464-8 PMC9126943 35606349

[68] M. Klotzsche , M. Vogel , S. Sachs , et al., “How Tobacco (Nicotiana Tabacum) BY‐2 Cells Cope with Eu(iii) – a Microspectroscopic Study,” The Analyst 148 (2023): 4668–4676. 37646162 10.1039/d3an00741c

[69] M. Vogel , R. Steudtner , T. Fankhänel , J. Raff , and B. Drobot , “Spatially Resolved Eu(III) Environments by Chemical Microscopy,” The Analyst 146 (2021): 6741–6745. 34570845 10.1039/d1an01449h

[70] B. Drobot , A. Bauer , R. Steudtner , et al., “Speciation Studies of Metals in Trace Concentrations: The Mononuclear Uranyl(VI) Hydroxo Complexes,” Analytical Chemistry 88 (2016): 3548–3555. 26977534 10.1021/acs.analchem.5b03958

[71] D. S. Smith , “Solution of Simultaneous Chemical Equilibria in Heterogeneous Systems: Implementation in Matlab,” n.d.

[72] S. P. Skinner , R. H. Fogh , W. Boucher , T. J. Ragan , L. G. Mureddu , and G. W. Vuister , “CcpNmr AnalysisAssign: A Flexible Platform for Integrated NMR Analysis,” Journal of Biomolecular NMR 66 (2016): 111–124. 27663422 10.1007/s10858-016-0060-y PMC5095159

[73] D. A. Case , R. E. Duke , R. C. Walker , et al., AMBER 22 (University Of California, 2022).

[74] P. Li , L. F. Song , and K. M. Merz Jr. , “Parameterization of Highly Charged Metal Ions Using the 12‐6‐4 LJ‐Type Nonbonded Model in Explicit Water,” The Journal of Physical Chemistry. B 119 (2015): 883–895. 25145273 10.1021/jp505875v PMC4306492

