# Supplementary material for: An Integrative Approach Toward Bio‐Inspired Sequestration of Rare Earth Elements
Source: ChemSusChem. 2026 Aug 12;19(16):e70900. doi: 10.1002/cssc.70900 (PMC13464047; doi:10.1002/cssc.70900)
Supplement: Supplementary file 1 [file CSSC-19-e70900-s001.pdf]

Supporting Information: An Integrative Approach Towards Bio-inspired Sequestration of Rare Earth Elements

Yulia Perevedentseva# [a], Lukas Waurick# [b], Dini Marlina[a], Gerda Techert[c], Yannic Müllers [a,d], Franziska Lederer[c], Heiko M. Möller[a], Michael U. Kumke[a], Ulrich Glebe* [a,d], Björn Drobot* [b]

[a] Institute of Chemistry, University of Potsdam, Karl-Liebknecht-Str. 24–25, 14476 Potsdam-Golm, Germany

[b] Institute of Resource Ecology, Helmholtz-Zentrum Dresden-Rossendorf, Bautzner Landstr. 400, 01328 Dresden, Germany

[c] Helmholtz Institute Freiberg for Resource Technology, Helmholtz-Zentrum Dresden-Rossendorf, Bautzner Landstr. 400, 01328 Dresden, Germany

[d] Fraunhofer Institute for Applied Polymer Research IAP, Geiselbergstr. 69, 14476 Potsdam-Golm, Germany

*correspondence: b.drobot@hzdr.de and ulrich.glebe.1@uni-potsdam.de

Experimental Section

The EF-hand 4 loop from CaM, DIDGDGQVNYEE (EF4), was commercially obtained for ITC and TRLFS spectroscopic studies. For NMR experiments, ^{15}N-labeled and ^{15}N,^{13}C-labeled peptides were expressed and purified as described in the Supplementary Information. The immobilized EF4 was in-house synthesized by solid-phase peptide synthesis (SPPS). We have chosen the lanthanides Eu^{3+}, Sm^{3+}, Tb^{3+}, La^{3+}, and Lu^{3+} for our study.

Isothermal Titration Calorimetry (ITC)

ITC measurements were performed using MicroCal PEAQ-ITC, Malvern Panalytical, Kassel, Germany. The measurement was carried out in triplicate, for which we used three different peptide concentrations, so that differences in complex formation could be observed well. For this, stock solutions of 750 µM $EuCl_3$ as well as 100 µM, 50 µM and 33 µM EF4 (each in 100 mM KCl, pH 6.0) were prepared. The sample cell was equilibrated with 100 mM KCl and then filled with the prepared EF4 solutions using a clean glass syringe. The ITC syringe was as well equilibrated with 100 mM KCl before being loaded with the $EuCl_3$ stock solution. The reference cell contained water (Aqua tridest.).

The following instrumental setup was applied: a total of 19 injections with a time interval of 150 seconds, with a lower volume of 0.4 µl for the first injection than for the other 18 injections with a volume of 2 µl each, a reference power of 7.00 µcal*s^{-1} and initial delay of 60 s. The measurement was carried out at a temperature of 25 °C and a stirring speed of 750 rpm. Additionally, a background measurement (control) was performed at the same settings, in which 750 µM $EuCl_3$ was titrated to 100 mM KCl (pH 6.0).

Data analysis was performed in Matlab, using a similar approach as previously described.[1] A spline based baseline subtraction followed by a singular value decomposition noise reduction was performed prior the data analysis. The analysis itself is based on a speciation calculation tool[2] modified in a way to optimize thermodynamic constants using the optimization toolbox in Matlab. The replicates of the titration with different peptide concentrations were simultaneously analyzed (global fitting) and parameter errors were estimated using a Monte Carlo approach, in which data were re-analyzed (100 times) after the addition of synthetic noise.

Time-Resolved Laser Fluorescence Spectroscopy (TRLFS)

Sample preparation

Ln^{3+} stock solutions (~0.1 M) were prepared from $LnCl_3 \cdot 6\ H_2O$; Ln = Eu, Sm, and Tb (Sigma Aldrich) dissolved in deionized water. Synthesized EF4 peptide was obtained from Peptides&Elephants (p&e). The peptide stock solution was prepared by weighing the amount according to the required concentration (~50 – 100 µM) and dissolved in MilliQ water. The stock solutions were stored at 4 °C before use. The concentration of Ln^{3+} and peptide stock solutions were determined using a UV/Vis spectrophotometer (Perkin Elmer, Lambda 750). For the titration experiment, both Ln^{3+} and EF4 solution were adjusted to pH around 5 by adding small amounts of $HClO_4$ (0.01 M) and/or NaOH (0.01 or 0.1 M). pH measurements were carried out using a pH meter (inoLab pH 720) with an electrode (Thermo scientific, ROSS inner filling solution).

Titration experiments

A titration series was carried out by titrating the Ln^{3+} with EF4. The Ln^{3+} concentration was 1 µM for Eu^{3+} or Tb^{3+} and 10 µM for Sm^{3+}. The Ln^{3+} solution was placed in a PMMA cuvette (optical length of 1 cm). To prevent Ln^{3+} dilution, 1 or 10 µM of the Ln^{3+} was added to the EF4. The EF4 was added stepwise. For the reverse titration, 10 µM EF4 was placed in the cuvette and titrated with Eu^{3+} containing 10 µM of EF4 to avoid dilution. After addition of titrant, the solution was mixed using a shaker (Vortex Genie 2, Scientific Industries) for 10 s. The luminescence was monitored at a room temperature.

The TRLFS measurements were carried out with a pulsed Nd:YAG laser (Quanta Ray, Spectra Physics, with repetition rate 20 Hz. The excitation wavelength for Eu^{3+}, Tb^{3+}, and Sm^{3+} was set to 394, 377 and 362 nm, respectively. The detector coupled to the 20 Hz laser is iCCD camera (iStar DH734 – 18H – 13, Andor technology) with a spectrograph (Shamrock 303i-A, SR0275, Andor technology) equipped with a 300 lines/mm grating (blaze: 500 nm).

The boxcar technique was used to measure the time resolved spectra. The initial delay time and other setting parameters are given in Table S1. The luminescence spectra were corrected for the spectral sensitivity of the grating and the iCCD camera. PARAFAC (parallel factor analysis) algorithm was used to deconvolute spectra, delay times, concentration depend distribution, and dissociation constant (K_D) of each species from the luminescence data. The analysis was performed in MATLAB R2022b (MathWorks) as described elsewhere.[3] As constraints, non-negativity was chosen on the spectral dimension while a speciation constrain was applied to the distribution dimension, and the luminescence decays of each species in the time dimension have been constrained to monoexponential decays.

NMR experiments

Plasmid constructs

For cloning, amplifying and expression, His_6-SUMO tag-EF4 was chosen.

Sequence of His_6-SUMO tag-EF4:

MGHHHHHHGSDSEVNQEAKPEVKPEVKPETHINLKVSDGSSEIFFKIKKTTPLRRLMEAFAKRQGKEMDSLRFLY
DGIRIQADQTPEDLDMEDNDIIEAHREQIGGDIDGDGQVNYEE

The His_6-SUMO tag-EF4 fragment was created using a PCR reaction. Primer design was carried out in the program SnapGene.

Primer sequence for cloning:

5'- gtcaggttaactacgaagaaTGAGATCCGGCTGCTAAC-3'

3' -cgtcaccgtcgatgtcCCCACCAATCTGTTCTCTG-5'

As the reaction's result, a fragment including His$_6$-SUMO tag was cut out of the vector pET-24a (Novagen) and a fragment of EF4 was added to His$_6$-SUMO tag. The resulting fragment was assembled with vector pET28 derivative (Novagen) using sticky ends. Golden Gate Assembly is accomplished by simultaneous digestion with restriction enzymes BsaI (New England Biolabs) and ligation by a T4 DNA ligase (New England Biolabs). The resulting vector pET28M-His$_6$-SUMO tag-EF4 was transformed into competent *Escherichia coli* Tuner (DE3) cells (Novagen) and amplified. Competent *E. coli* cells were transformed with 5 ng of plasmid. Transformed cells were selected on LB media agar plates (10 g/L tryptone, 5 g/L yeast extract, 10 g/L NaCl, 10 mM glucose and 0.5 mg/ml kanamycin). A single colony was used to inoculate 20 ml of LB medium containing 200 µL glucose and 20 µL antibiotic. The culture was incubated 12 h at 37 °C and 160 rpm. 920 µL of the culture was added to 80 µL DMSO and stored at -80 °C until the peptide was expressed.

Expression

The M9 media consisted of 17.1 mM NaCl, 22 mM KH_2PO_4, 42 mM Na_2HPO_4, 18.3 mM $^{15}NH_4Cl$ pH 7.4, ^{13}C-glucose 11mM, vitamins x500 mix 2ml/L, kanamycin 50 µg/mL, CEL TONE ^{15}N ^{13}C 100 mg/L, 2 mM $MgSO_4$, 0.1 mM $CaCl_2$, 0.1 mM $FeCl_3$, 0.1 mM $MnCl_2$. $^{15}NH_4Cl$, ^{13}C-glucose and CEL TONE $^{15}N^{13}C$ used as the only source of nitrogen and carbon. The cells were initially grown in 20 ml LB medium and then amplified in 1L M9 media. The culture was shaken at 37 °C and 100 rpm to an OD_{600} ~0.7-0.9 and was then induced by 0.5 mM IPTG at 37 °C for 16 hours. The cells were harvested by centrifugation at 10000 g, 4°C, 20 min and frozen at -80 °C. Normal yield for double labelled sample is 2 g per 1L.

Purification

All purification steps were performed at 4 °C. 1 ml protease inhibitors was added in 10 ml Buffer A (50 mM TRIS, 100 mM NaCl, pH 8.0). In order to remove large DNA fragments released upon cell rupture 0.1 ml of 0.5 M $MgCl_2$ and 0.1 M $CaCl_2$ and a spatula tip of DNAse were added. Wet weight cells were suspended in buffer. Afterwards, cells were ruptured 5 minutes through the high-pressure homogenizer (Emulsiflex-C3, Avestin, Ottawa, Canada) at 20000 bar. The lysate was centrifuged for 15 min at 100.000g at 4 °C and filtered. A Ni-Sepharose Fast Flow column (GE Healthcare, Freiburg, Germany) on Äkta Purifier (Cytiva, Wilmington, United States) fast protein liquid chromatography system was washed with water and equilibrated with buffer A and 4% buffer B (50 mM TRIS, 100 mM NaCl, 500 mM imidazol, pH 8.0). The pre-filtered supernatant was loaded onto the column. After washing with buffer A, bound protein was eluted in a step gradient with buffer B. Fractions with SUMO-peptide were concentrated using 3 kDa MWCO Amicon Ultra (Sigma Aldrich). ULP1 protease was used for cleavage of the His$_6$-SUMO tag from EF-hand 4. 1 mg ULP1 protease per 15 mg peptide was added to the sample and left for 16 h at room temperature. Afterwards, the solution was passed through 3 kDa MWCO Amicon Ultra. Filtrate was used for the next assays.

NMR measurements

All NMR measurements were performed on a Bruker NEO 500 MHz and Avance III 600 MHz spectrometer equipped with N_2 cooled cryoprobes (Bruker Biospin GmbH, Rheinstetten, Germany). For processing and analysing the spectra, TopSpin 4.09 (Bruker) and CCPNMR[4] was used. NMR samples were prepared in 30 mM MES, 150 mM NaCl buffer, 5 % D_2O, 4mM NaN_3, 0.1 mM DSS, pH 5.2. The backbone and sidechain assignment were obtained by combination of following spectra: 2D ^{15}N HSQC, ^{13}C HSQC, TOCSY, COSY and 3D HNCaCb, HNCa, HNCaCO, HNCO, HNCOCa, ^{15}N TOCSY. Assignment was performed using CCPNMR software. Chemical shifts are listed in Table S2. To determine the pKa values of aspartic and glutamic acids, the 0.1 mM ^{13}C-^{15}N EF4 samples were measured in the pH range between 1.75 – 7.4 at 25°C. Two dimensional H2(Cα)CO type experiment was used for determination of sidechain carboxylic carbon. The analogue of the Henderson-Hasselbalch (HH) equation was used as a fitting model for pKa calculation (Equation 2).

$$\delta_{obs} = \delta_0 + \frac{\Delta\delta}{1+10^{[pH-pKa]}} \quad (2)$$

where $\Delta\delta$ is the pH-shift that shows the chemical shift perturbations.

Ln titration was performed under the following conditions: Peptide concentration was 100 µM, and the Ln concentration (La^{3+}, Lu^{3+}, Sm^{3+}, Eu^{3+} and Ce^{3+}) was varied from 5 to 2000 µM. ^{15}N HSQC experiments were

recorded for all samples. The CSP values were obtained using the TOPSPIN program by peak deconvolution using mixed lineshape. The temperature experiments ^{1}H-NOESY were carried out in the temperature range 4 °C – 25 °C (Tm=500 ms). The highest signal intensity was observed for temperature 4°C. For NOE measurements of the La^{3+}-EF4 complex, Ln ions were added to EF4 solution to ratio 1:1. The peptide's flexibility was assessed via ^{1}H-^{15}N heteronuclear NOE measurements and ^{1}H-^{13}C-HSQC spectra. For ^{1}H-^{15}N heteronuclear NOE, the relaxation delay was 3 s and the temperature 25 °C. Concentrations of samples for ^{1}H-^{15}N heteronuclear NOE measurements were next: for free EF4 c=1 mM, for 1:1 La^{3+}-EF4 complex c_{La3+}=0.9 mM, c_{EF4}=0.85 mM, for 2:1 La^{3+}-EF4 complex c_{La3+}=10 mM, c_{EF4}=0.2 mM.

Molecular Dynamics (MD) Simulations

MD simulations were performed as solvent simulations without any surface using the program package Amber 22.[5] The ff14SB force field is applied to the EF4 of CaM. The peptide was created as a string without any restraints from its amino acid sequence in a deprotonation state observed in pH = 7. Simulations were performed with La^{3+}, Sm^{3+} and Eu^{3+} added automatically by using a Coulombic potential on a grid or manually with coordination to D5 or D3. Simulations with automatically positioned Ln^{3+} were performed with Eu^{3+} and with Eu^{3+} and Sm^{3+} as a complex with two Ln^{3+}. For the starting condition of the coordination to D5, simulations were performed for La^{3+}, Sm^{3+} and Eu^{3+}. Simulations with coordination to D3 were performed for La^{3+}. In case of setting the Ln^{3+} position automatically, this step was done before adding the solvent to avoid steric interactions with water molecules to have influence on the positioning. Also, in this case the Ln^{3+} is positioned close to E12. 12-6-4-Lennard-Jones-type parameters, developed by Metz *et al.*, were used for La^{3+}, Sm^{3+} and Eu^{3+}.[6] Each peptide was then soaked in a periodic octahedral box filled with water molecules, resulting in a minimal layer thickness of 10 Å. In addition, for simulations with one Ln^{3+}, Na^{+} ions were added to the system to neutralize the overall charge. In the case of two Ln^{3+}, Cl^{-} were used to do so.

Immobilization of EF4 peptide

Peptide immobilization was conducted through standard fluorenylmethoxycarbonyl (Fmoc)-based solid phase peptide synthesis on a MultiPep RSi automatic synthesizer (Intavis, Tübingen, Germany). As matrix, 100 µmol Tentagel resin (0.43 mmol/g) with 4-hydroxymethylbenzoic acid (HMBA) linker (Rapp Polymere GmbH, Tübingen, Germany) was used per reactor. First, Fmoc-glycine was coupled to the resin utilizing 3.9 eq. 2-(1H-benzotriazol-1-yl)-1,1,3,3-tetramethyluronium hexafluorophosphate (HBTU), 4 eq. amino acid, 8 eq. N-methylmorpholine (NMM) and 0.1 eq. 4-dimethylaminopyridine (DMAP). This step was repeated three times for 2 h. Subsequently, capping of eventually remaining functional groups of the resin (without linked glycine) were capped by treatment with acetic anhydride / pyridine (1:1 V/V) for 30 min. The resin was washed with DMF (3 x 1 ml) and, if not immediately continued, DCM (5 x 1 ml). Cleavage of the N-terminal Fmoc protection group was conducted through a mixture of 20% piperidine and 5% formic acid in DMF (1 x 3 min, 1 x 17 min). The next amino acids (Carbolution Chemicals GmbH, St. Ingbert, Germany) were coupled via standard procedure using 3.9 eq. HBTU, 4 eq. amino acid and 8 eq. NMM (1 x 60 min). After each coupling step, the resin was treated with 0.85 eq. acetic anhydride in DMF (1 x 30 min). Side chain protection groups were removed by treating the resin with a mixture of TFA/TIPS/water (95:2.5:2.5) for 2 hours. After that, the resin was washed with DMF (3 x 1 ml), MilliQ water (3 x 1 ml), 100 mM acetate buffer (pH 5) (3 x 1 ml), MilliQ water (3 x 1 ml), DMF (3 x 1 ml), DCM (5 x 1 ml) and then dried in vacuum.

For analysis, peptides were cleaved off by treating the resin with 1 ml 500 mM NaOH (aq.) / MeCN (1:1 V/V) for 20 min. The filtrate was collected and lyophilized to obtain the peptide. The purity of EF4 was determined by reverse phase HPLC. Measurements were performed using an Agilent 1200 system (Agilent Technologies Inc., Santa Clara, USA), equipped with a degasser, autosampler, multi-wavelength DAD detector and automated fraction collector. The column used for analytical runs was a Brownlee SPP2.7 (C18, 2.1x150 mm, PerkinElmer, Waltham, USA). The solvent system was water (0.1% formic acid) and MeCN (0.1% formic acid) with a flow rate of 0.3 mL/min. Solvent purity was HPLC gradient grade or higher. For instrument control and data collection, ChemStation B03.02 (Agilent Technologies Inc., Santa Clara, USA) was used.

WILEY-VCH

Method for EF4: 10-90% MeCN within 15 min, linear gradient, isocratic 90% MeCN for 6 min, 10% MeCN for 9 min for re-equilibration.

Sequence identity (EF4-G) was verified by ESI-MS. The analyses were performed using an Agilent 6230C TOF LC/MS (Agilent Technologies) equipped with an ESI source operating in positive ion mode. The instrument was controlled using SingleQuad 2.2. Before the measurement, the sample was dissolved in MilliQ water. All spectra were recorded in positive ion mode.

Determination of grafting density

The grafting density of the assembled EF4-G peptide was analyzed by cleavage of the Fmoc-protection group after the last amino acid coupling. 10 mg resin was incubated with 20% piperidine in DMF (1 mL). The suspension was shaken for 20 minutes and the supernatant was removed. The concentration of the dibenzofulven adduct was determined by an absorption measurement at 301 nm on a SPECORD 210 spectrophotometer (Analytik Jena) using an extinction coefficient of 7800 M^{-1} cm^{-1}.

Evaluation of linker stability

10 mg resin with fully assembled EF4-G and N-terminal Fmoc-protection group was incubated with 1 ml of either 10 mM HCl (pH 2), 100 mM acetate buffer (pH 5) or 100 mM phosphate buffer (pH 7.4) for 5 days. The suspension was shaken twice per day for 30 minutes. After that, the resin was washed with MilliQ water (3 x 1 ml), DMF (3 x 1 ml) and DCM (5 x 1 ml). Peptide loading was determined by Fmoc cleavage as described before.

Lanthanide accumulation by immobilized EF4

Resin (Tenta) with EF4-G, EF4-G with protected carboxylic groups and the resin itself were incubated with Sm^{3+} at pH 6 in a 1:1 ratio (100 µM EF4 : 100 µM $SmCl_3$). To this end, 8.4 mg resin (peptide loading: 177.7 µmol/g, corresponding to 100 µM EF4 in 15 ml) was suspended in 15 ml of the respective Sm^{3+} solution. The suspensions were shaken for 24 h at room temperature. Afterwards, the suspensions were centrifuged (30 min / 13000 g) and the supernatant was collected (triplicate) for ICP-MS analysis. After careful removal of the supernatant, the remaining resin was acidified using 1 ml of a 1 M HNO_3 and centrifuged again. This sample was analyzed by ICP-MS as well.

ICP-MS analysis

Elemental analysis was carried out on a Quadrupol-ICP-MS-Systeme (iCap RQ, Thermo Fisher Scientific, 1550 W, argon) using an internal Sc standard. Measurements were the mean of technical triplicates.

Chemical microscopy of EF4-Tenta particle

Chemical microscopy was carried out on a LabRAM system (Horiba Jobin Yvon, Lyon, France), equipped with a 532 nm laser for excitation. Data acquisition and analysis was performed as described elsewhere.[7] A total of 528 spectral scans were performed with a spatial resolution of ~10 µm in x and y direction (220 x 240 µm).

WILEY-VCH

Supplementary results

Table S1: Summary and comparison of the binding parameters along the binding models for ITC with different complexes as well as the three different lanthanide ions Eu^{3+}, Sm^{3+} and Tb^{3+}.

Number of complexes	Stoichiometry Eu^{3+}:EF4	Binding parameter	Eu^{3+}	Sm^{3+}	Tb^{3+}
1	1 : 1	K_D (µM)	9.55	9.66	9.52
		ΔH (kJ*mol^{-1})	24.67	20.84	25.44
		n	1.19	1.05	1.05
2	1 : 1	K_D (µM)	6.79	6.90	9.52
		ΔH (kJ*mol^{-1})	25.04	21.74	28.41
	2 : 1	K_D (µM)	98.91	115.20	83.75
		ΔH (kJ*mol^{-1})	39.27	31.27	36.83
		n	0.95	0.84	0.82
	1 : 1	K_D (µM)	9.49	9.54	20.99
		ΔH (kJ*mol^{-1})	27.05	20.49	32.71
	1 : 2	K_D (µM)	97.61	57.28	139.90
		ΔH (kJ*mol^{-1})	18.09	19.59	22.51
		n	1.17	1.18	1.00
3	1 : 1	K_D (µM)	9.43	10.41	11.30
		ΔH (kJ*mol^{-1})	25.69	21.10	24.23
	2 : 1	K_D (µM)	170.80	65.81	254.30
		ΔH (kJ*mol^{-1})	21.51	19.77	38.22
	1 : 2	K_D (µM)	352.50	962.70	844.80
		ΔH (kJ*mol^{-1})	39.06	23.57	51.99
		n	1.13	1.14	1.08
K_D…dissociation constant; ΔH…reaction enthalpy; n… calculated stoichiometry value for the 1:1 complex					

WILEY-VCH

Table S2: Residue assignment of EF4.

Seq. number	Residue	Atom ID	Atom type	Isotope number	Chemical shift. ppm
1	ASP	HA	H	1	4.289
		C	C	13	172.580
		CA	C	13	53.364
		CB	C	13	39.933
		CG	C	13	178.817
2	ILE	N	N	15	120.414
		H	H	1	8.650
		HA	H	1	4.222
		HB	H	1	1.878
		HG12	H	1	1.192
		HG13	H	1	1.462
		HG2	H	1	0.923
		HD1	H	1	0.870
		CA	C	13	61.445
		CB	C	13	38.759
		CG1	C	13	20.417
		CG2	C	13	17.604
		CD1	C	13	13.228
		C	C	13	175.754
3	ASP	N	N	15	123.848
		H	H	1	8.434
		HA	H	1	4.633
		HB2	H	1	2.734
		HB3	H	1	2.687
		C	C	13	176.624
		CA	C	13	54.347
		CB	C	13	40.860
		CG	C	13	179.950
4	GLY	N	N	15	109.512
		H	H	1	8.301
		HA	H	1	3.976
		C	C	13	174.240
		CA	C	13	45.541
5	ASP	N	N	15	8.294
		H	H	1	4.644
		HA	H	1	2.740
		HB	H	1	176.941
		C	C	13	54.230
		CA	C	13	40.886
		CB	C	13	180.146
		CG	C	13	8.294
6	GLY	N	N	15	109.160
		H	H	1	8.390
		HA2	H	1	3.929
		HA3	H	1	3.959

		C	C	13	174.220
		CA	C	13	45.453
7	GLN	N	N	15	119.915
		H	H	1	8.185
		HA	H	1	4.351
		HB2	H	1	1.995
		HB3	H	1	2.083
		HE21	H	1	7.565
		HE22	H	1	6.832
		HG	H	1	2.312
		C	C	13	175.873
		CA	C	13	55.667
		CB	C	13	29.345
		CG	C	13	33.861
		CD	C	13	180.653
		NE	N	15	112.732
8	VAL	N	N	15	121.496
		H	H	1	8.162
		HA	H	1	4.052
		HB	H	1	1.948
		HG1	H	1	0.876
		HG2	H	1	0.764
		C	C	13	175.376
		CA	C	13	62.159
		CB	C	13	32.775
		CG1	C	1	20.417
		CG2	C	1	21.042
9	ASN	N	N	15	122.605
		H	H	1	8.406
		HA	H	1	4.726
		HB	H	1	2.781
		HD21	H	1	7.555
		HD22	H	1	6.899
		C	C	13	174.542
		CA	C	13	52.761
		CB	C	13	39.122
		CG	C	13	177.059
		ND	N	15	112.977
10	TYR	N	N	15	121.655
		H	H	1	8.222
		HA	H	1	4.586
		HB2	H	1	2.945
		HB3	H	1	3.085
		HD	H	1	7.123
		HE	H	1	6.821
		C	C	13	175.374
		CA	C	13	57.903
		CB	C	13	38.892
11	GLU	N	N	15	122.750

WILEY-VCH

		H	H	1	8.237
		HA	H	1	4.328
		HB2	H	1	1.907
		HB3	H	1	2.066
		HG	H	1	2.259
		C	C	13	175.049
		CA	C	13	56.020
		CB	C	13	30.182
		CG	C	13	35.577
		CD	C	13	183.623
12	GLU	N	N	15	126.813
		H	H	1	7.918
		HA	H	1	4.111
		HB2	H	1	1.913
		HB3	H	1	2.060
		HG	H	1	2.271
		C	C	13	180.840
		CA	C	13	57.684
		CB	C	13	30.541
		CG	C	13	35.890
		CD	C	13	183.975

Table S3: pKa values from pH NMR titration of all Asp and Glu in EF4.

Residue	pKa from H2(Ca)CO	pKa from ^{13}C-HSQC
D1	3.44 ± 0.05	3.43 ± 0.097
D3	4.20 ± 0.03	4.21 ± 0.03
D5	4.15 ± 0.03	4.21 ± 0.03
E11	4.62 ± 0.03	4.63 ± 0.03
E12	4.63 ± 0.03	4.64 ± 0.03

Table S4: Starting conditions of each MD simulation run of $[Ln(EF4)]^{2-}$complexes with La^{3+}, Eu^{3+} and Sm^{3+}

MD run	Metal	Stoichometry (M : L)	Metal positioning
1	La^{3+}	1 : 1	D5
2	La^{3+}	1 : 1	D5
3	La^{3+}	1 : 1	D5
4	Sm^{3+}	1 : 1	D5
5	Sm^{3+}	1 : 1	D5
6	Sm^{3+}	1 : 1	D5
7	Eu^{3+}	1 : 1	D5
8	Eu^{3+}	1 : 1	D5
9	Eu^{3+}	1 : 1	D5
10	La^{3+}	1 : 1	D3

WILEY-VCH

11	La^{3+}	1 : 1	D3
12	La^{3+}	1 : 1	D3
13	Sm^{3+}	2 : 1	D5 + E12/C-Terminus
14	Sm^{3+}	2 : 1	D5 + E12/C-Terminus
15	Sm^{3+}	2 : 1	D5 + E12/C-Terminus
16	Eu^{3+}	2 : 1	D5 + E12/C-Terminus
17	Eu^{3+}	2 : 1	D5 + E12/C-Terminus
18	Eu^{3+}	2 : 1	D5 + E12/C-Terminus

Table S5: Average coordination numbers for the last 30 ns of each MD simulation run of $[Ln(EF4)]^{2-}$ complexes with La^{3+}, Eu^{3+} and Sm^{3+}; ion positioning at the start of the simulation close to D5.

MD run	Metal	M – H_2O	M –	M –	M –	M – E11	M – E12	M –
1	La^{3+}	6.66	0	0	1.03	1.52	0	0
2	La^{3+}	6.57	0	1.25	1.19	0	0.18	0
3	La^{3+}	5.93	0	0.99	0.99	0	0.99	0
4	Sm^{3+}	6.91	0	0	1.00	0	0	0.99
5	Sm^{3+}	4.94	0	0	0.99	1.98	0	0.99
6	Sm^{3+}	6.87	0	0	0.99	0	0	1.03
7	Eu^{3+}	6.00	0	0.58	0.99	0	1.63	0
8	Eu^{3+}	6.38	0.69	0	0.99	0.84	0	0
9	Eu^{3+}	6.60	0.14	0	0.99	0	1.17	0

Table S6: Average coordination numbers for the last 30 ns of each MD simulation run of $[La(EF4)]^{2-}$; ion positioning at the start of the simulation close to D3.

MD run	Metal	M – H2O	M –	M –	M –	M –	M –	M –
10	La^{3+}	5.15	0	1.80	0.99	0	1.73	0
11	La^{3+}	6.89	0	1.87	1.00	0	0	0
12	La^{3+}	4.42	1.00	1.27	0	1.05	0.11	1.08

Table S7: Average coordination numbers for the last 30 ns of each MD simulation run of $[Ln_2(EF4)]^{+}$ complexes with $Sm^{3+}$and Eu^{3+}; ion positioning at the start of the simulation by algorithm; D1 moves close to the metal but there is no stable coordination.

MD run	Metal	M – H2O	M – D1	M – D3	M – D5	M – E11	M – E12	M – C-
13	Sm^{3+} 1	5.92	0	0	0	0.99	0.99	0.99
13	Sm^{3+} 2	6.44	0	0.99	0.99	0	0	0
14	Sm^{3+} 1	4.94	0.44*	0	0	1.43	1.09	0.99
14	Sm^{3+} 2	5.93	0	1.43	1.52	0	0	0
15	Sm^{3+} 1	5.64	0	0	0	1.27	0.99	0.99
15	Sm^{3+} 2	6.26	0	1.35	1.06	0	0	0
16	Eu^{3+} 1	5.83	0	0	0	1.09	0.99	0.99
16	Eu^{3+} 2	6.86	0.99	1.05	0	0	0	0
17	Eu^{3+} 1	5.92	0	0	0	0.99	0.99	0.99
17	Eu^{3+} 2	6.86	1.04	1.00	0	0	0	0
18	Eu^{3+} 1	5.93	0	0	0	0.99	0.99	0.99
18	Eu^{3+} 2	5.08	0	1.44	1.37	0	0	0

Table S8: Conditions used during evaluation of linker stability. Percentage of residual peptide was determined in respect to untreated Fmoc-EF4-resin.

Conditions	Loading [µmol/g]	Residual Peptide
dry	177.7	
100 mM phosphate buffer (pH 7.4), 5 days	177.4	99.8 %
100 mM acetate buffer (pH 5), 5 days	178.1	100.3 %
10 mM HCl (pH 2), 5 days	177.8	100.1 %
1 M HNO_3	173.1	97.4 %

Isothermal titration calorimetry

Based on the baseline-corrected thermograms, we obtained several fit curves and tested their robustness using the Monte Carlo method. For the sole occurrence of a 1:1 complex, the superimposed data points do not fit the fitting curve properly (Figure S1 (B)). For this reason, the raw data were again fitted assuming the sequential formation of two (Figure S1 (D), Figure S2 (F)) or three (Figure S2 (H)) complexes in order to improve the robustness of the fit curves.

WILEY-VCH

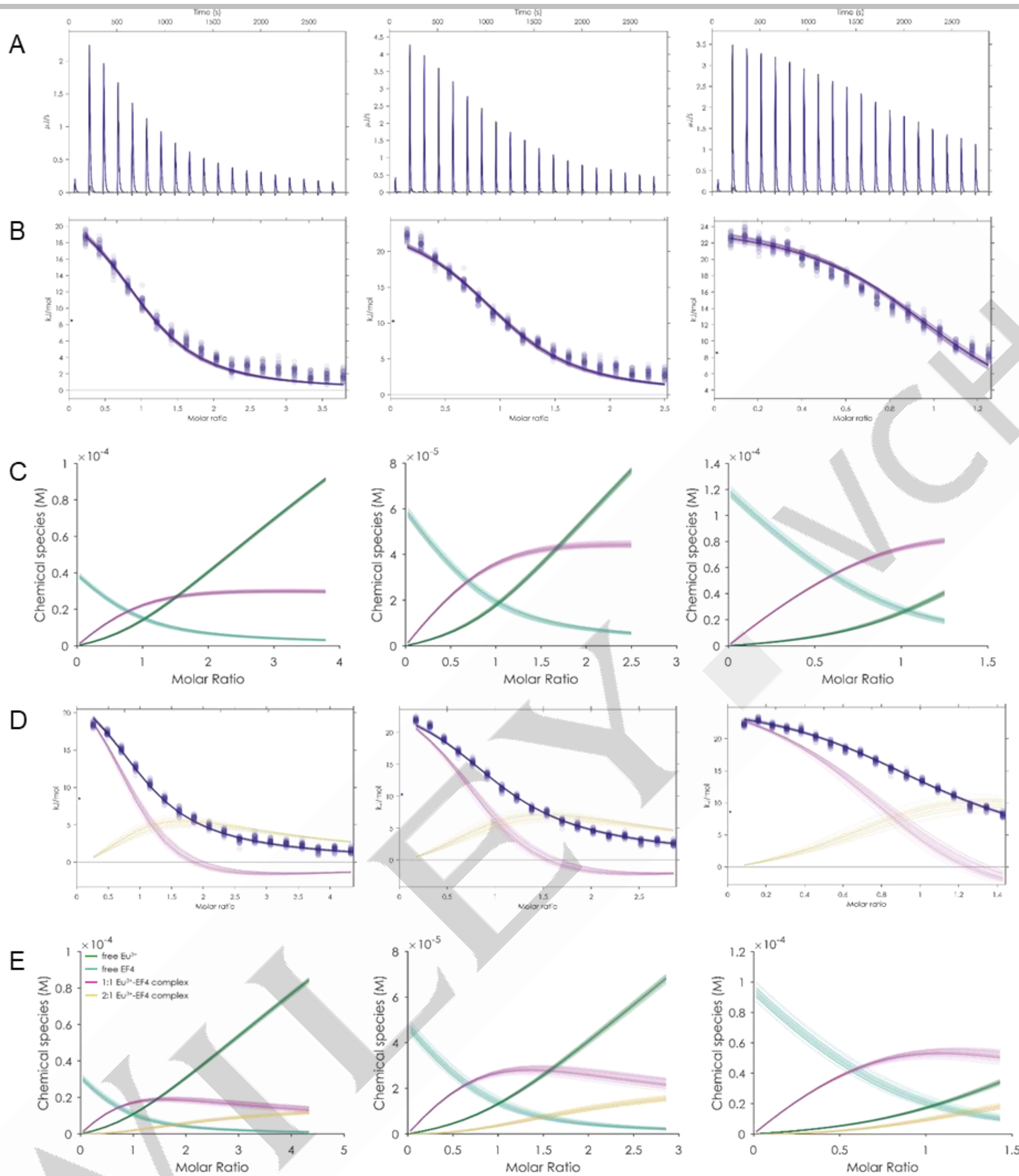

Figure S1. ITC of Eu^{3+} titration to EF4. **(A)** Baseline-corrected thermograms of titrations of 750 µM Eu^{3+} each to either 33 µM (left), 50 µM (middle) or 100 µM (right) EF4 (blue) at pH 6.0 and the corresponding background measurement (black). **(B)** Raw data fit curves (line) derived from thermograms and robustness test using Monte Carlo method with 100 generated data sets overlaid (symbols) assuming an one-complex binding model with a 1:1 Eu^{3+} -EF4 complex for all three EF4 concentrations (same order as for (A)). **(C)** Speciations resulting from (B). **(D)** Same as for (B) assuming a two-complex binding model with a 1:1 and 2:1 Eu^{3+} -EF4 complex. **(E)** Speciations resulting from (D).

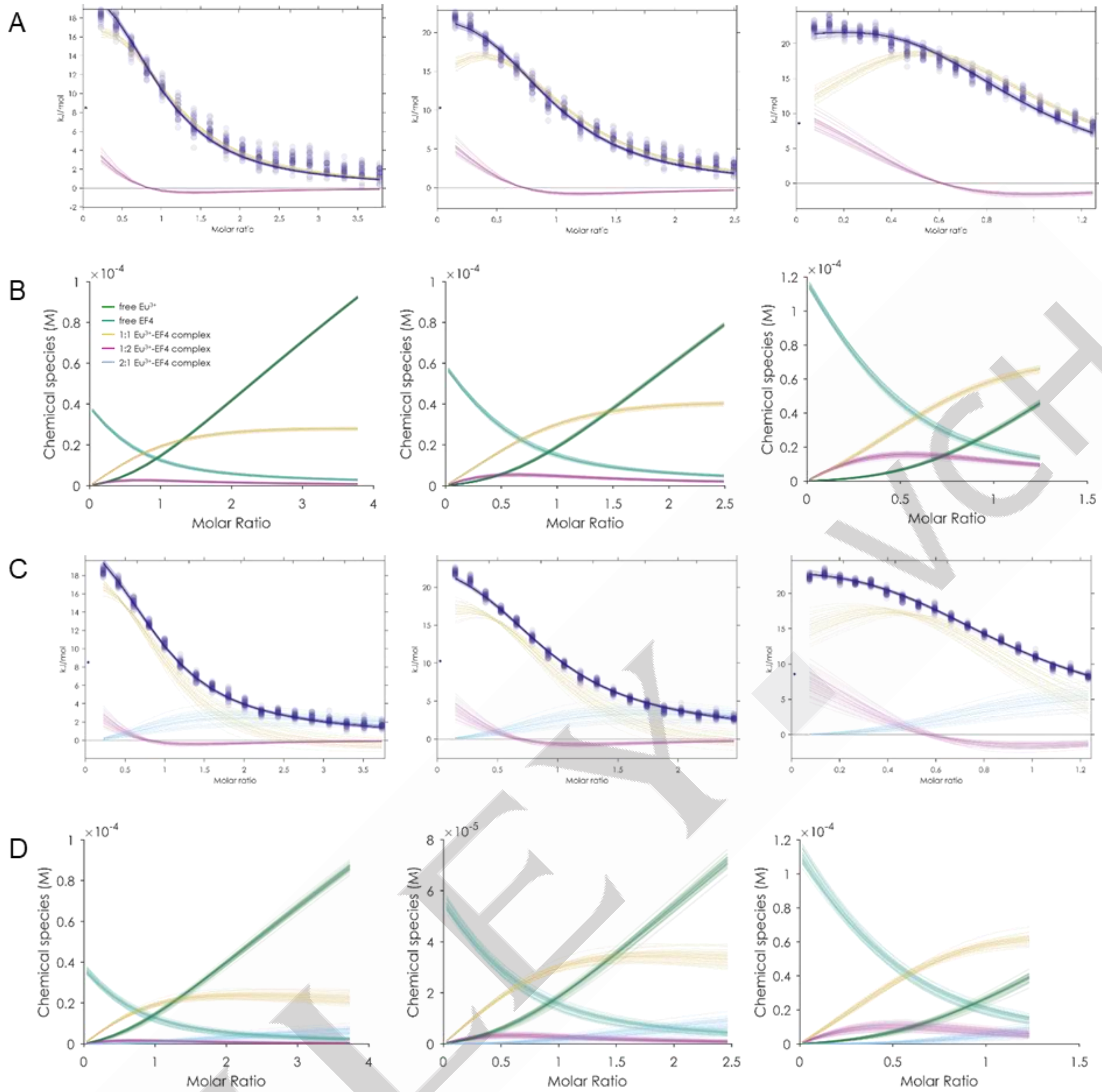

Figure S2. ITC of Eu^{3+} titration to EF4. **(A)** Raw data fit curves (line) derived from thermograms and robustness test using Monte Carlo method with 100 generated data sets overlaid (symbols) assuming a two-complex binding model with a 1:1 and 1:2 Eu^{3+} -EF4 complex for all three EF4 concentrations (same order as for Fig. S1 (A)). **(B)** Speciations resulting from (A). **(C)** Same as for (A) assuming a three-complex binding model with a 1:1, 2:1 and 1:2 Eu^{3+}-EF4 complex**. (D)** Speciations resulting from (C).

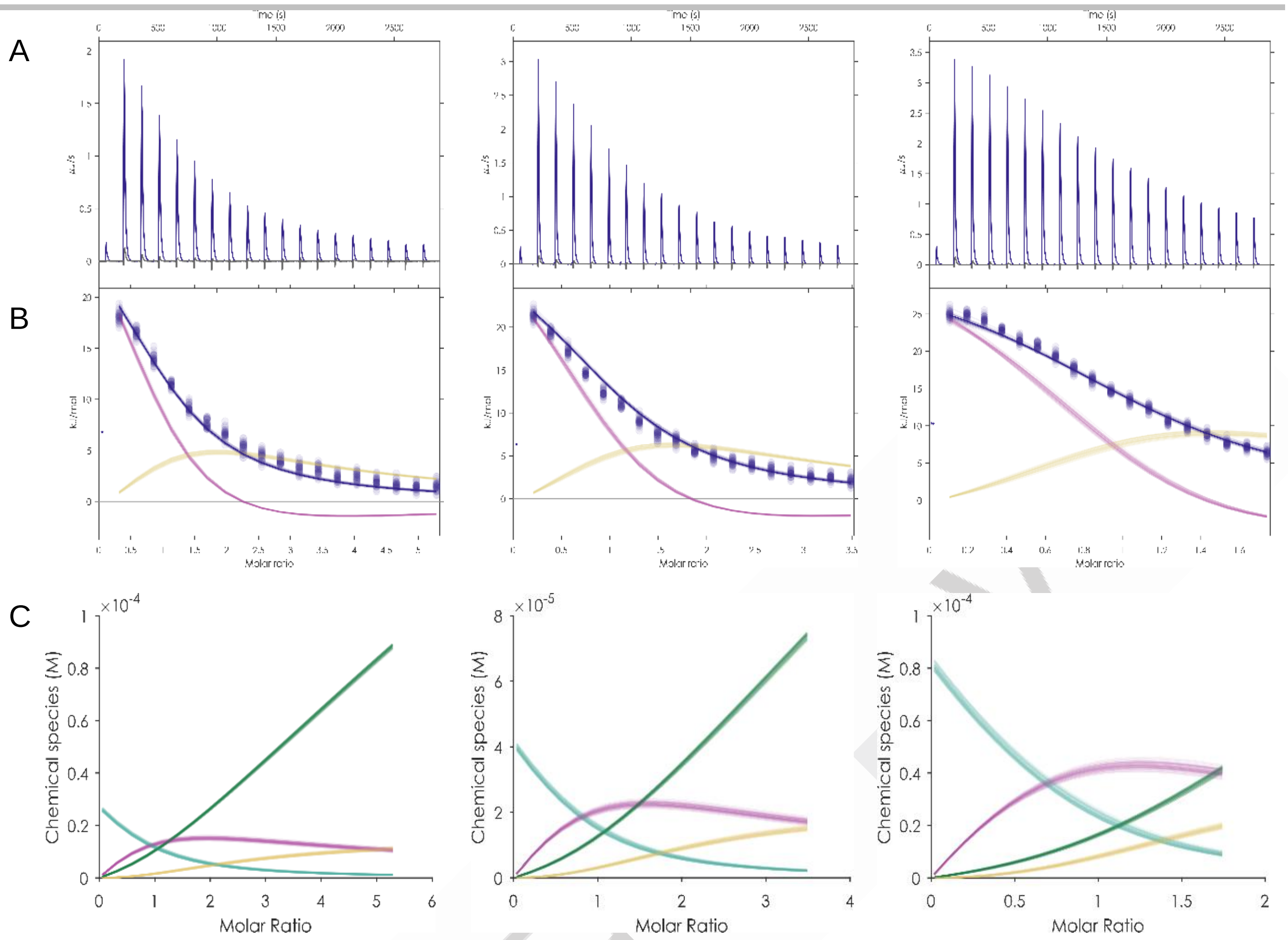

Figure S3. ITC of Tb^{3+} titration to EF4. **(A)** Baseline-corrected thermograms of titrations of 750 µM Tb^{3+} each to either 33 µM (left), 50 µM (middle) or 100 µM (right) EF4 (blue) at pH 6.0 and the corresponding background measurement (black). **(B)** Raw data fit curves (line) derived from thermograms and robustness test using Monte Carlo method with 100 generated data sets overlaid (symbols) assuming a two-complex binding model with a 1:1 and 2:1 Tb3+-EF4 complex for all three EF4 concentrations (same order as for (A)). **(C)** Speciations resulting from (B).

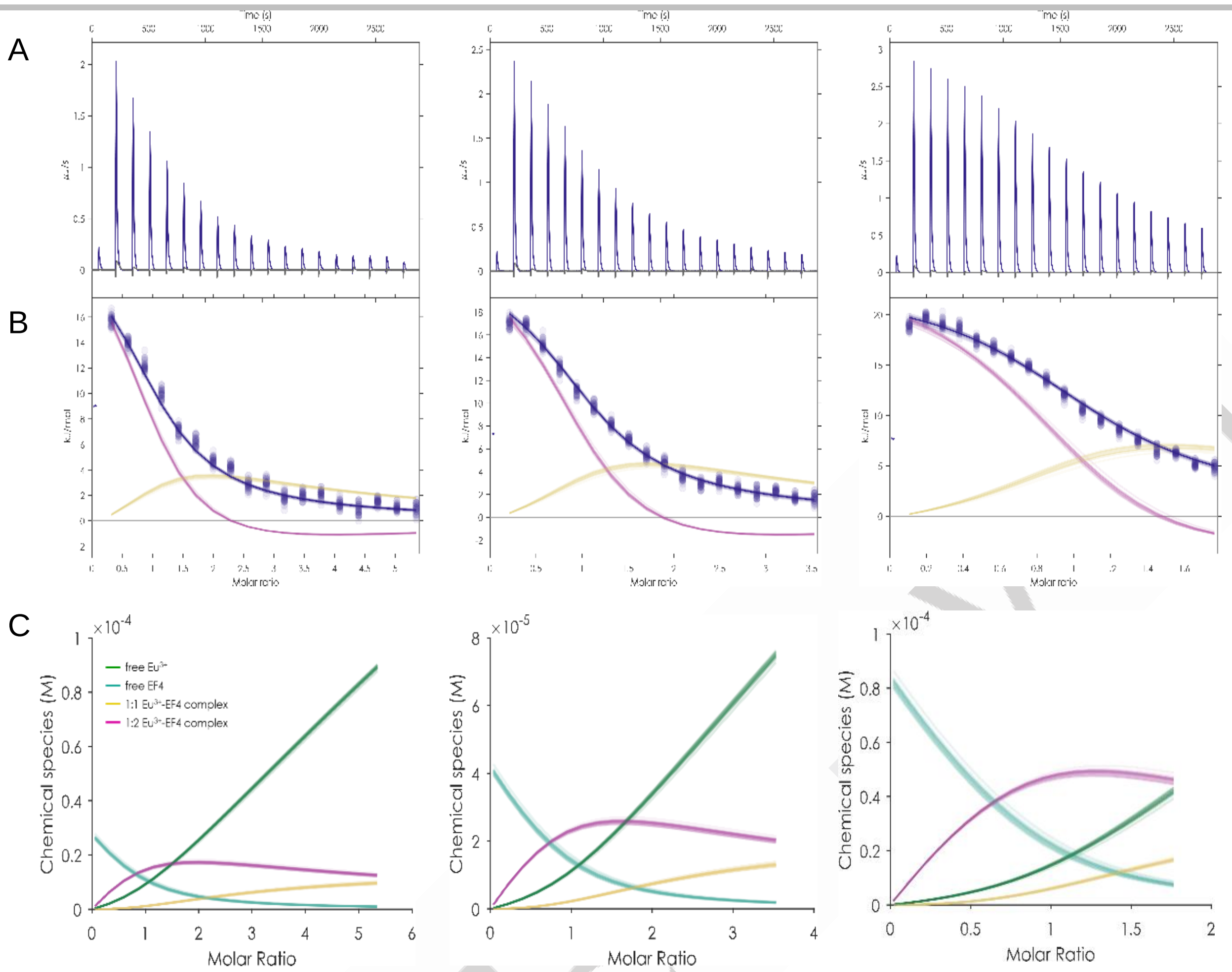

Figure S4. ITC of Sm^{3+} titration to EF4. **(A)** Baseline-corrected thermograms of titrations of 750 µM Sm^{3+} each to either 33 µM (left), 50 µM (middle) or 100 µM (right) EF4 (blue) at pH 6.0 and the corresponding background measurement. **(B)** Raw data fit curves (line) derived from thermograms and robustness test using Monte Carlo method with 100 generated data sets overlaid (symbols) assuming a two-complex binding model with a 1:1 and 2:1 Sm^{3+}-EF4 complex for all three EF4 concentrations (same order as for (A)). **(C)** Speciations resulting from (B).

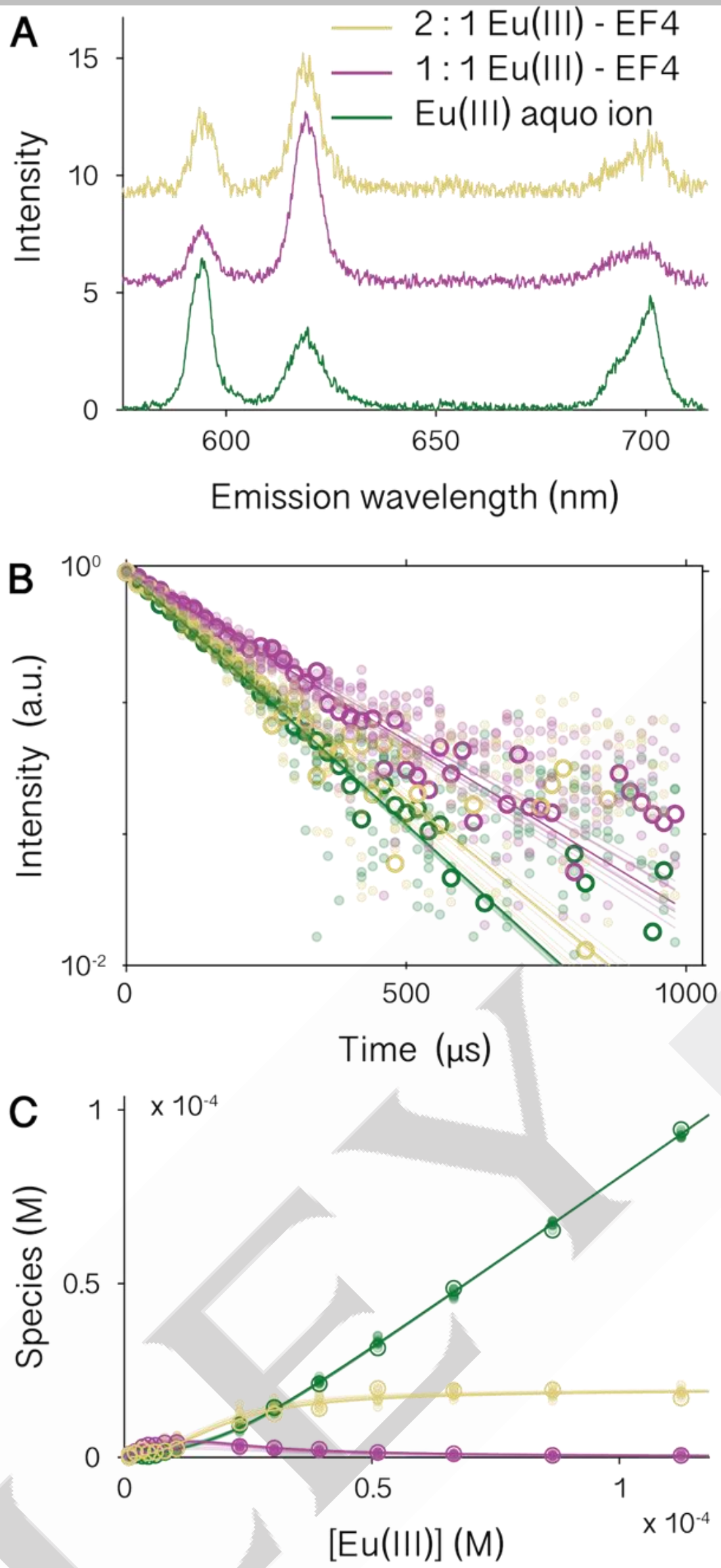

Figure S5. PARAFAC deconvolution of TRLFS data where 10 µM EF4 was titrated with Eu^{3+} 0 – 112 µM. **(A)** Luminescence spectra of each Eu^{3+} species (color code: green = free Eu^{3+} aquo ion, magenta = 1:1 Eu^{3+} - EF4, yellow = 2:1 Eu^{3+} -EF4 complex), **(B)** luminescence decay kinetics, and **(C)** speciation

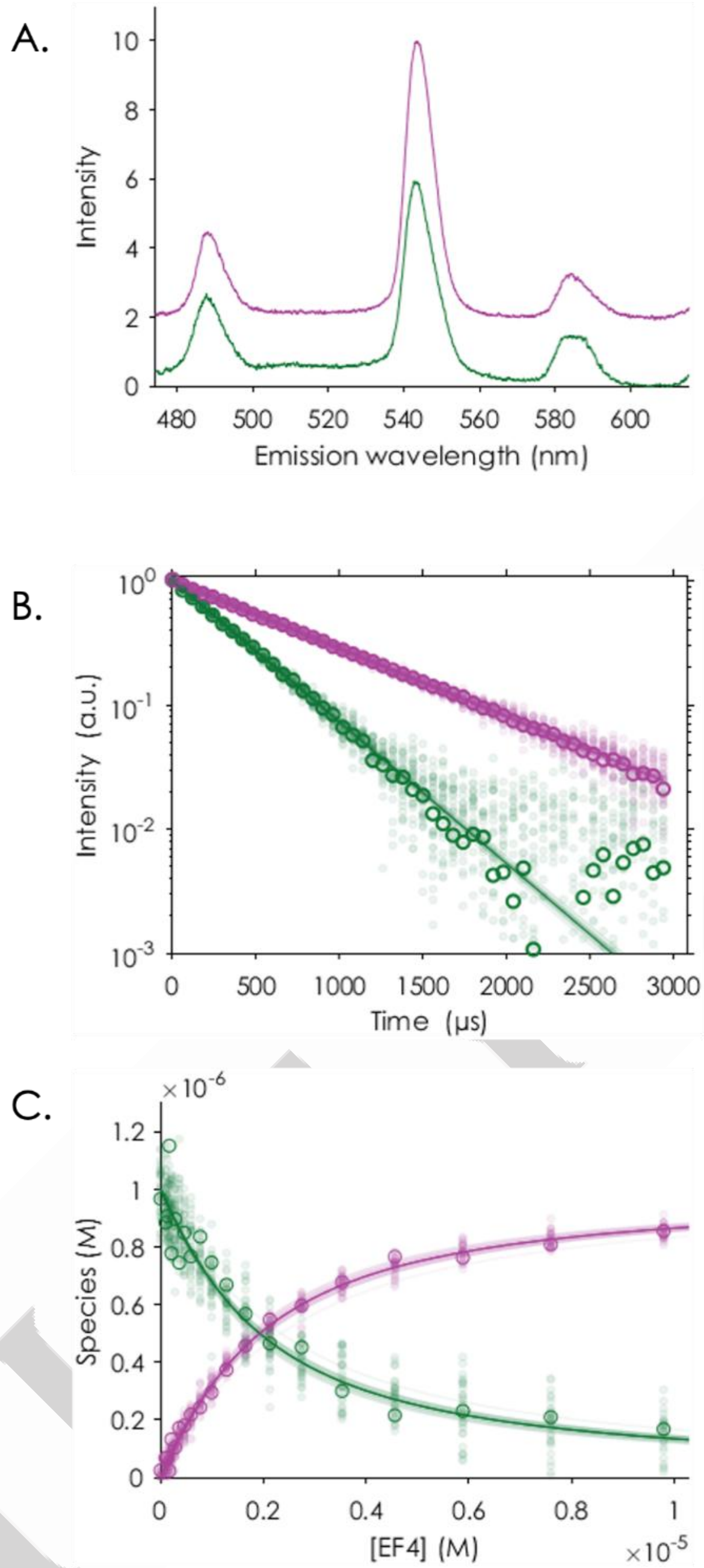

Figure S6. PARAFAC deconvolution where 1 µM of Tb^{3+} was titrated with EF4 0 - 10 µM **(A)** deconvoluted spectra, **(B)** decay time of observed species (Tb^{3+} aquo ion = 381 µs, 1:1 Tb^{3+} -EF4 complex = 793 µs), **(C)** speciation.

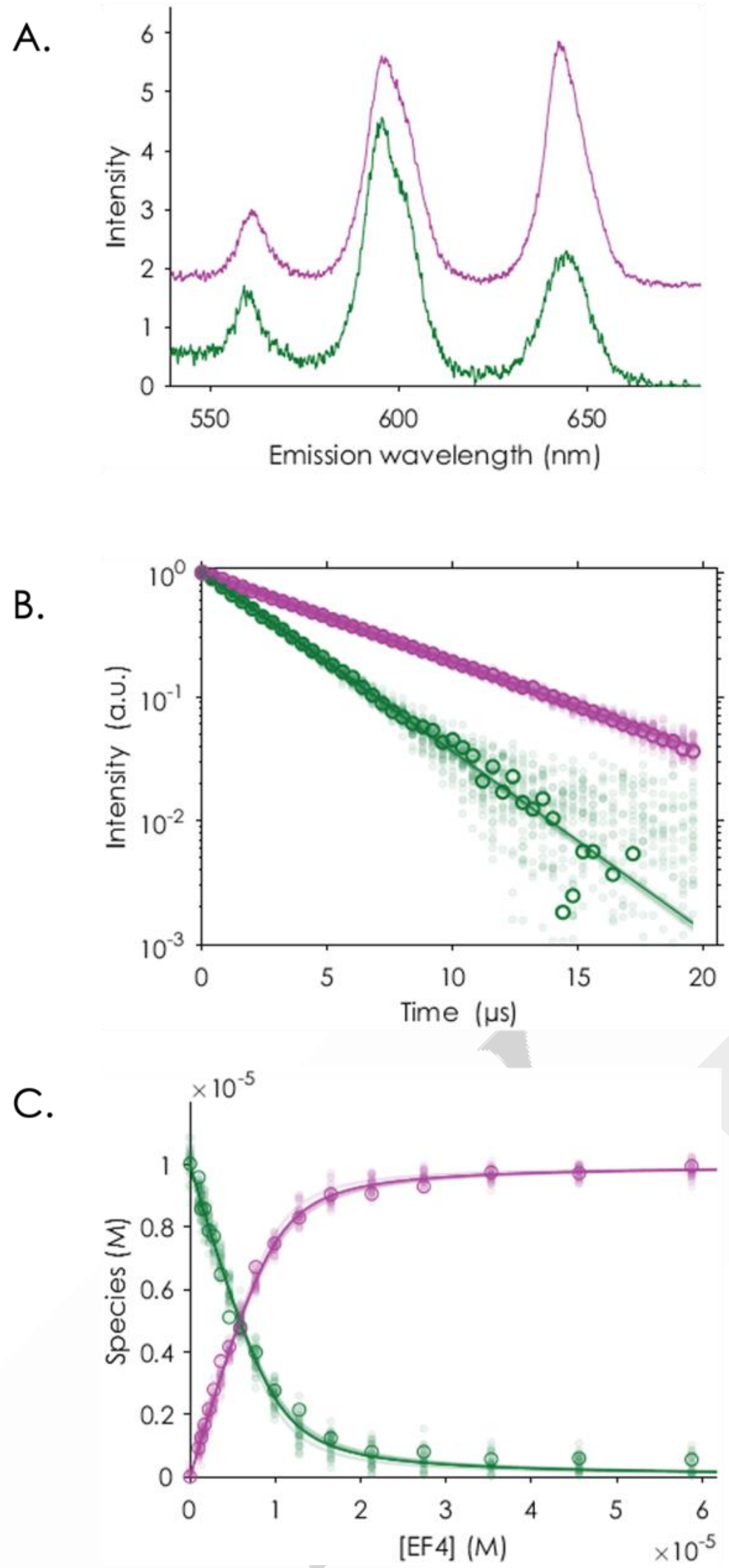

Figure S7. PARAFAC deconvolution where 10 µM of Sm^{3+} was titrated with EF4 0 - 59 µM. **(A)** deconvoluted spectra, **(B)** decay time of observed species (Sm^{3+} aquo ion = 3 µs, 1:1 Sm^{3+}-EF4 complex = 6 µs, **(C)** speciation.

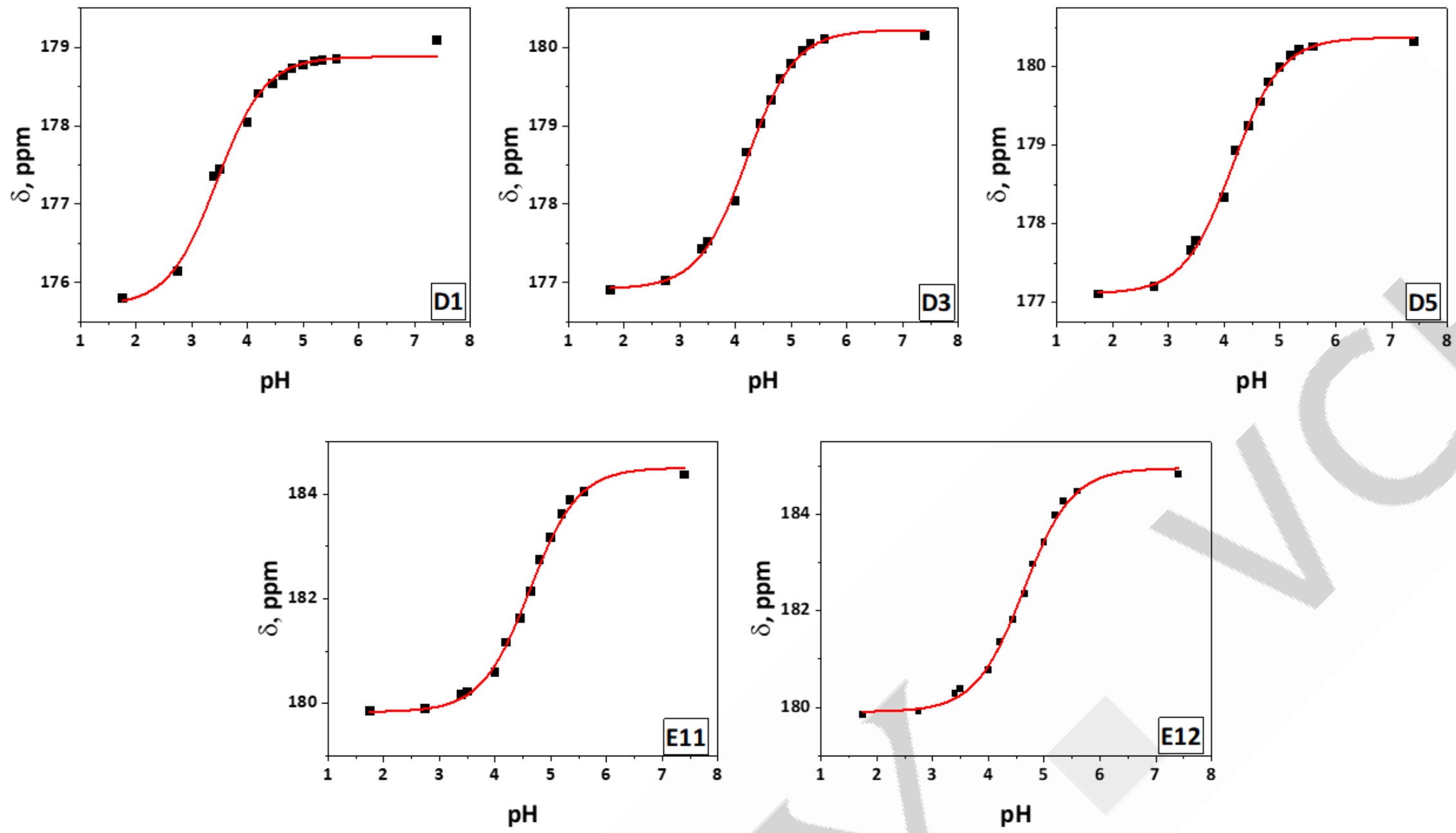

Figure S8. Titration curves of pH-dependent side-chain CO chemical shifts of 3 Asp residues **(A-C)** and the 2 Glu residues **(D-E)** from EF4 in pH range 1.75-7.4. pKa values were calculated using standard Henderson-Hasselbalch equation (see Table S3).

WILEY-VCH

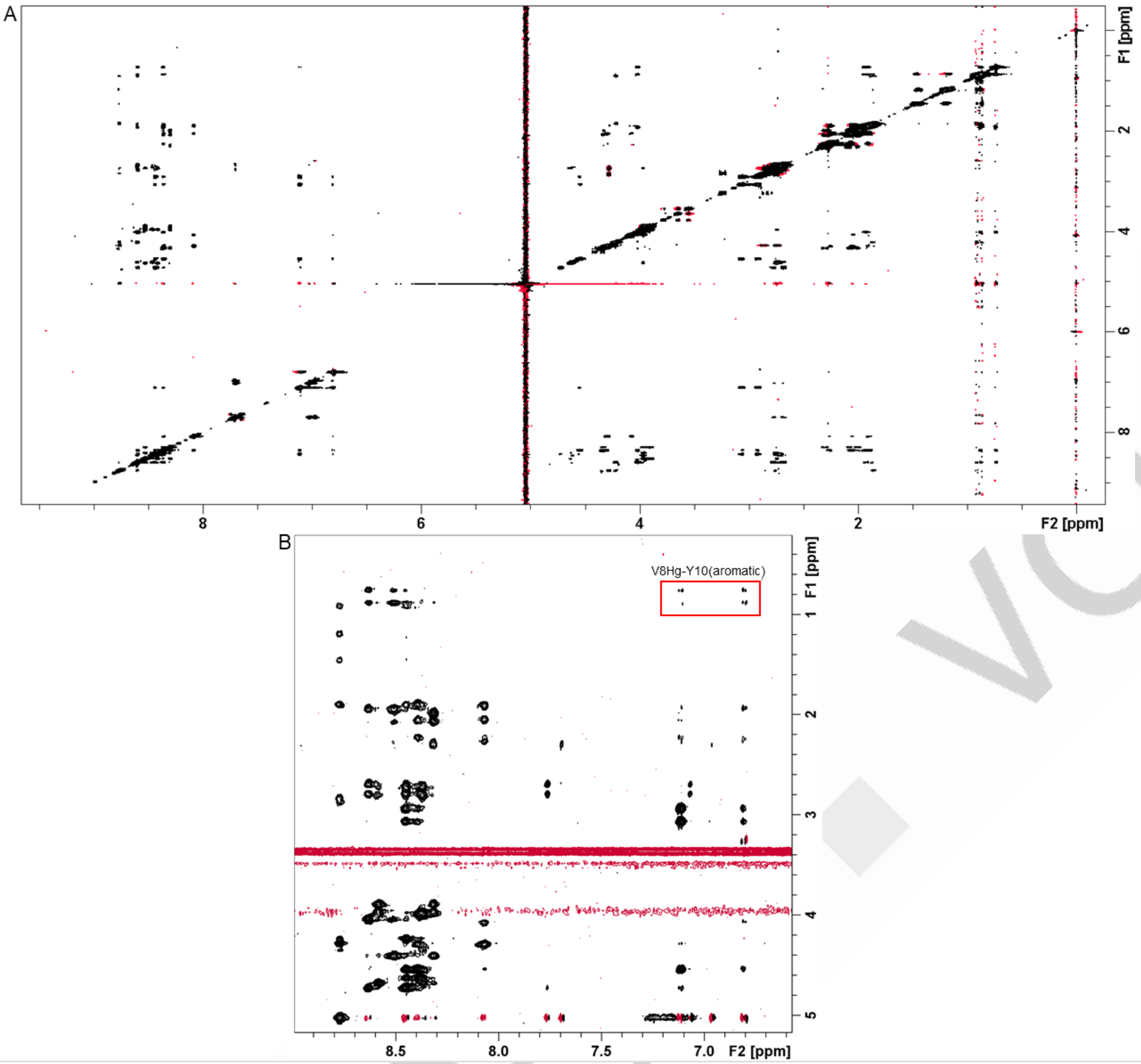

Figure S9. NOESY spectra of La^{3+}-EF4 complex **(A)** and amide region of the same spectra **(B)**. Spectra measured at 600 MHz magnetic fields and temperature 25°C. Concentrations of EF4 and La^{3+} were both 0.5 mM. The NOE cross-peaks between valine (V8) and tyrosine (Y10) are highlighted in the red box.

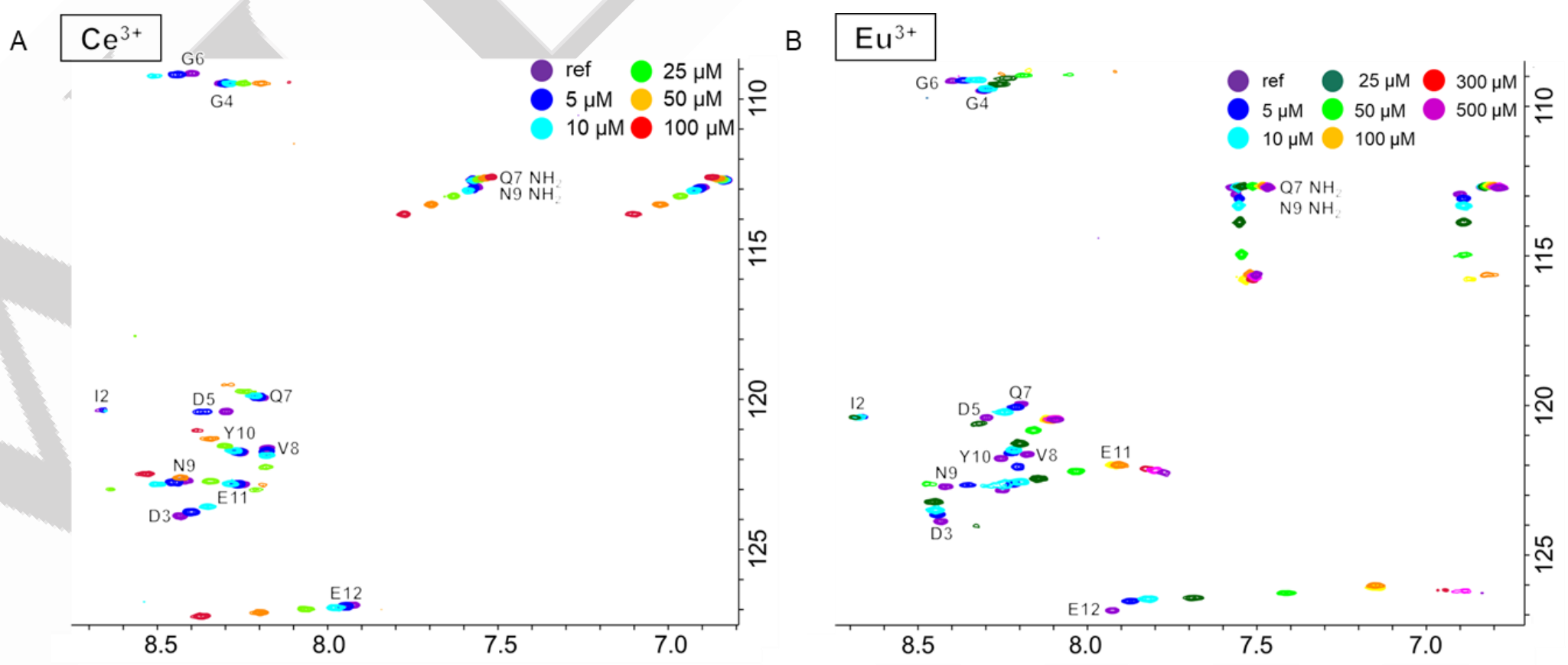

Figure S10. ^{1}H-^{15}N-HSQC titration data of 100 µM ^{15}N EF4 with Ce^{3+} **(A)** and Eu^{3+} **(B)**. Lanthanide concentration increases from violet to red.

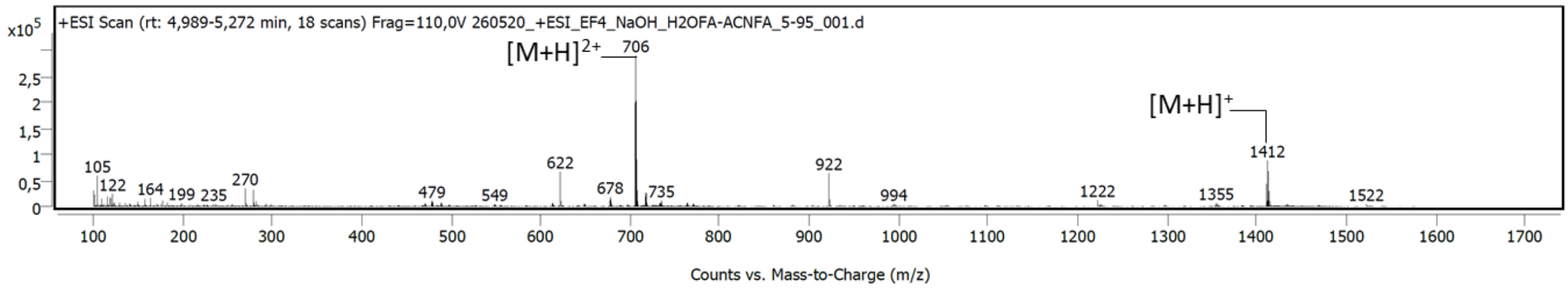

Figure S11. ESI-MS measurement of EF4-G peptide cleaved from HMBA-Tentagel resin.

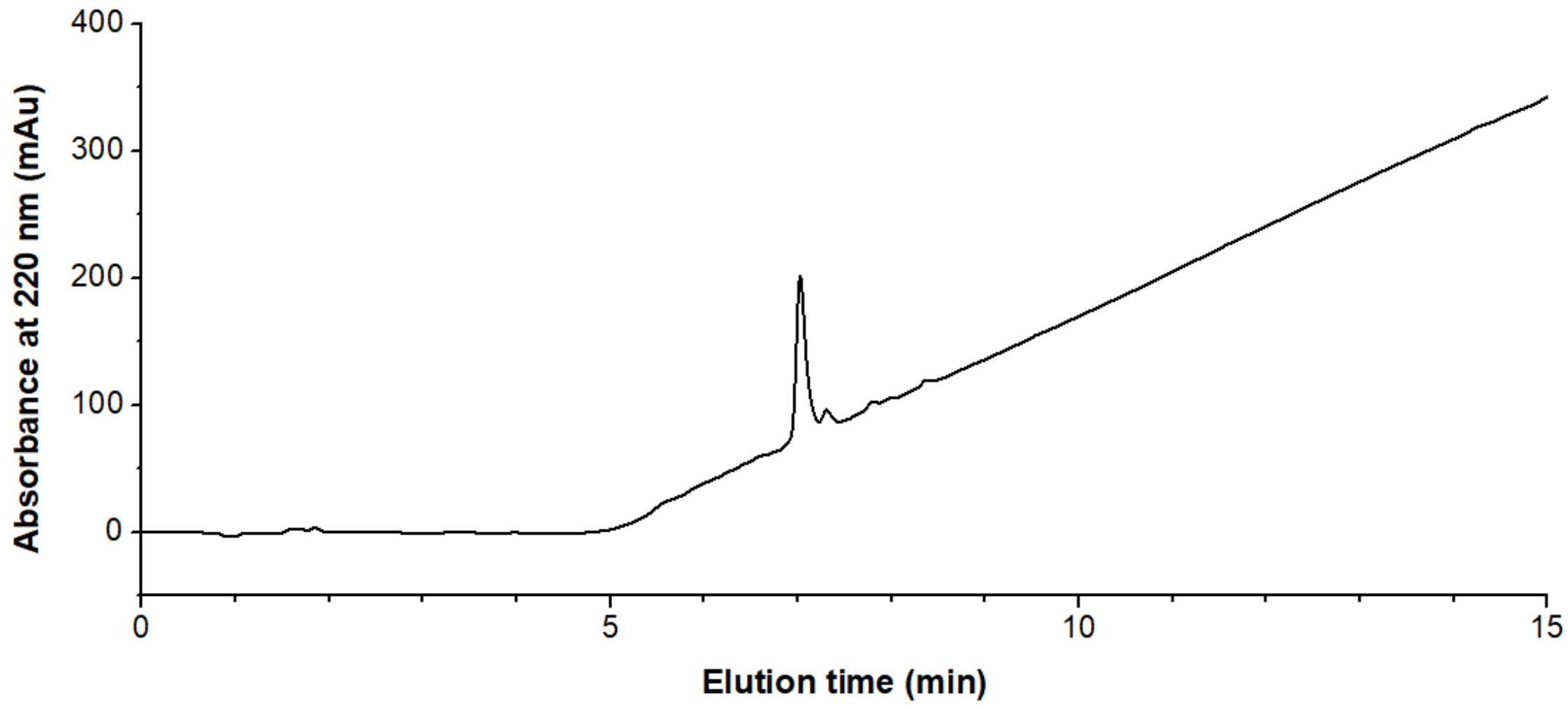

Figure S12. RP-HPLC elugram of EF4-G peptide cleaved from HMBA-Tentagel resin. Absorbance was measured at 220 nm.

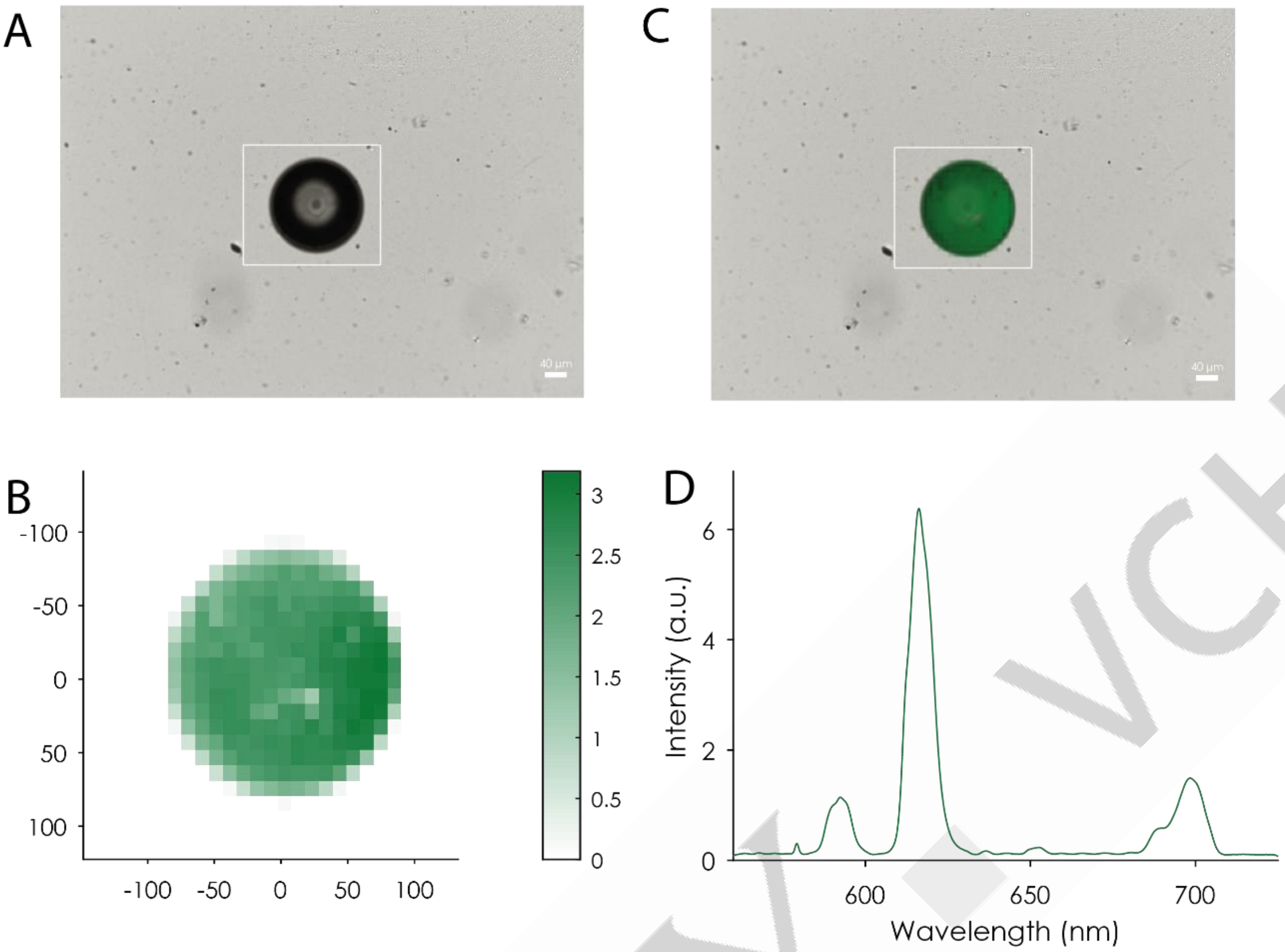

Figure S13. Chemical microscopy of a Tenta-EF4 particle incubated with Eu^{3+}. Optical image of the particle and the mapped region (ROI, frame) using a 10x objective (scale bar 40 µm) **(A)**. Spectral distribution within the ROI (10 µm resolution) **(B)**. Overlay of the optical image and the spectral distribution **(C).** Emission spectrum of Eu^{3+} within the spectral distribution **(D).**

References

[1] S. M. Gutenthaler, S. Tsushima, R. Steudtner, M. Gailer, A. Hoffmann-Röder, B. Drobot, L. J. Daumann, "Lanmodulin peptides – unravelling the binding of the EF-Hand loop sequences stripped from the structural corset" *Inorg Chem Front* **2022**, *9*, 4009–4021.
[2] D. S. Smith, "Solution of Simultaneous Chemical Equilibria in Heterogeneous Systems: Implementation in Matlab" **n.d.**
[3] B. Drobot, A. Bauer, R. Steudtner, S. Tsushima, F. Bok, M. Patzschke, J. Raff, V. Brendler, "Speciation Studies of Metals in Trace Concentrations: The Mononuclear Uranyl(VI) Hydroxo Complexes" *Anal. Chem.* **2016**, *88*, 3548–3555.
[4] S. P. Skinner, R. H. Fogh, W. Boucher, T. J. Ragan, L. G. Mureddu, G. W. Vuister, "CcpNmr AnalysisAssign: a flexible platform for integrated NMR analysis" *J. Biomol. NMR* **2016**, *66*, 111–124.
[5] D. A. Case, H. M. Aktulga, K. Belfon, I. Y. Ben-Shalom, J. T. Berryman, S. R. Brozell, D. S. Cerutti, T. E. C. III, G. A. Cisneros, V. W. D. Cruzeiro, T. A. Darden, N. Forouzesh, G. Giambasu, T. Giese, M. K. Gilson, H. Gohlke, A. W. Goetz, J. Harris, S. Izadi, S. A. Izmailov, K. Kasavajhala, M. C. Kaymak, E. King, A. Kovalenko, T. Kurtzman, T. Lee, P. Li, C. Lin, J. Liu, T. Luchko, R. Luo, M. Machado, V. Man, M. Manathunga, K. M. Merz, Y. Miao, O. Mikhailovskii, G. Monard, H. Nguyen, K. A. O'Hearn, A. Onufriev, F. Pan, S. Pantano, R. Qi, A. Rahnamoun, D. R. Roe, A. Roitberg, C. Sagui, S. Schott-Verdugo, A. Shajan, J. Shen, C. L. Simmerling, N. R. Skrynnikov, J. Smith, J. Swails, R. C. Walker, J. Wang, J. Wang, H. Wei, X. Wu, Y. Xiong, Y. Xue, D. M. York, S. Zhao, Q. Zhu, P. A. Kollman, *Amber 22*, University Of California, San Francisco, **2022**.
[6] P. Li, L. F. Song, K. M. Jr. Merz, "Parameterization of Highly Charged Metal Ions Using the 12-6-4 LJ-Type Nonbonded Model in Explicit Water" *J. Phys. Chem. B* **2015**, *119*, 883–895.
[7] M. Vogel, R. Steudtner, T. Fankhänel, J. Raff, B. Drobot, "Spatially resolved Eu(III) environments by chemical microscopy" *Analyst* **2021**, *146*, 6741–6745.

WILEY-VCH
